# Acute Biphasic Effects of Ayahuasca

**DOI:** 10.1371/journal.pone.0137202

**Published:** 2015-09-30

**Authors:** Eduardo Ekman Schenberg, João Felipe Morel Alexandre, Renato Filev, Andre Mascioli Cravo, João Ricardo Sato, Suresh D. Muthukumaraswamy, Maurício Yonamine, Marian Waguespack, Izabela Lomnicka, Steven A. Barker, Dartiu Xavier da Silveira

**Affiliations:** 1 Departamento de Psiquiatria, Universidade Federal de São Paulo, São Paulo, Brazil; 2 Centro de Matemática, Computação e Cognição, Universidade Federal do ABC, Santo André, Brazil; 3 Schools of Pharmacy and Psychology, University of Auckland, Auckland, New Zealand; 4 Departamento de Análises Clinicas e Toxicológicas, Faculdade de Ciências Farmacêuticas, Universidade de São Paulo, São Paulo, Brazil; 5 Department of Comparative Biomedical Sciences, School of Veterinary Medicine, Louisiana State University, Baton Rouge, Louisiana, United States of America; National Scientific and Technical Research Council (CONICET)., ARGENTINA

## Abstract

Ritual use of ayahuasca, an amazonian Amerindian medicine turned sacrament in syncretic religions in Brazil, is rapidly growing around the world. Because of this internationalization, a comprehensive understanding of the pharmacological mechanisms of action of the brew and the neural correlates of the modified states of consciousness it induces is important. Employing a combination of electroencephalogram (EEG) recordings and quantification of ayahuasca's compounds and their metabolites in the systemic circulation we found ayahuasca to induce a biphasic effect in the brain. This effect was composed of reduced power in the alpha band (8–13 Hz) after 50 minutes from ingestion of the brew and increased slow- and fast-gamma power (30–50 and 50–100 Hz, respectively) between 75 and 125 minutes. Alpha power reductions were mostly located at left parieto-occipital cortex, slow-gamma power increase was observed at left centro-parieto-occipital, left fronto-temporal and right frontal cortices while fast-gamma increases were significant at left centro-parieto-occipital, left fronto-temporal, right frontal and right parieto-occipital cortices. These effects were significantly associated with circulating levels of ayahuasca’s chemical compounds, mostly N,N-dimethyltryptamine (DMT), harmine, harmaline and tetrahydroharmine and some of their metabolites. An interpretation based on a cognitive and emotional framework relevant to the ritual use of ayahuasca, as well as it's potential therapeutic effects is offered.

## Introduction

Ayahuasca is the name of an amazonian vine, *Banisteriopsis caapi*, translated as "vine of the soul" [1]. It is also the name for concoctions prepared with the vine, which most frequently include other plants as additives, especially *Psychotria viridis* or *Diplopterys cabrerana* [2,3]. This powerful psychoactive brew was, and still is, largely used by Amerindian cultures for healing, divination and community bonding, among other uses [2,3]. More recently, it spread around the world with Brazilian churches like Santo Daime and União do Vegetal (UDV) [4].

Research into the chemical composition of ayahuasca shows the main active compounds in the plants and in the brew to be the beta-carbolines harmine, harmaline and tetrahydroharmine (present in *B*. *caapi*) and N,N-dimethyltryptamine (DMT, present in *P*. *viridis* and *D*. *cabrerana*) [5–9]. The beta-carbolines, especially harmaline, were first proposed as the main active compounds [10–12]. But later beta-carbolines' main function was hypothesized to be peripheral monoamine-oxidase (MAO) inhibition. This would allow DMT, a potent psychedelic compound [13], to access the brain [14]. Peripheral MAO inhibition by beta-carbolines in ayahuasca was evidenced using rat liver homogenates [15,16]. Later harmine, usually the most concentrated beta-carboline in ayahuasca samples, and harmaline, usually the less concentrated [9], were found to be 10,000-fold more potent at inhibiting MAO-A than MAO-B, with the difference obtained from extracts of *Banisteriopsis* reaching 2,500-fold greater potency for MAO-A [17]. Therefore, inhibition of MAO-A allows DMT to access the brain, rendering it orally psychoactive. DMT is then actively transported into the brain through an active three-step mechanism [18]. The required combination of DMT and harmine for psychoactivity after oral ingestion was further corroborated by human self-experiments [19,20]. In the brain, DMT acts mostly as a 5HT-2A receptor agonist [21], possibly also being the endogenous ligand for sigma-1 receptors [22] as well as other receptors such as the trace amine receptor [23].

The identification of the neural correlates of the modified state of consciousness induced by ayahuasca has proven challenging. Most studies to date employed the electroencephalogram (EEG) to record brain oscillatory activity after ayahuasca intake. The first was done in field conditions (Santo Daime religious ceremonies) before and after 45 to 60 minutes from ingestion of one dose. Results revealed increased power in the 36–44 Hz frequency band at left occipito-temporo-parietal electrodes, with tendencies to power decreases in theta and delta bands [24]. A subsequent EEG study was also done in field conditions (non-religious workshop) and reported increased alpha in the occipital lobes and increased theta also in frontal regions. However these recordings were done 4 to 6 hours after the initial dose, with subjects drinking up to three doses in the interim [25]. The third study was the first aiming control for the placebo effect through the use of a lyophilized ayahuasca administered in double-blind design in a laboratory environment. EEG was recorded continuously and revealed alpha power decrease at left-temporal and centro-parieto electrodes peaking 90 minutes after ingestion. Decreases were also reported for delta and theta in parietal areas and increases were observed in the beta band at central and parieto-temporal locations [26]. Another study with the lyophilizate employed low-resolution electromagnetic tomography (LORETA), revealing decreases in alpha-2 (10–12 Hz), delta and theta frequencies, with alpha power decrease located at the multisensory parieto-temporo-occipital junction [27]. A fifth study conducted with only two subjects recorded multiple times in open label sessions suggested ayahuasca increased gamma band coherence (36–44 and 50–64 Hz) in widespread regions of the scalp [28]. More recently, again using the lyophilizate double-blind methodology, it was reported that ayahuasca increased beta band power measured as the average spectrum of 19 electrodes recording from different brain regions. This effect was reported to increase after ingestion of a second dose [29].

Aiming at disentangling some of the contradictions from the previous literature to further understand the effects of ayahuasca in the oscillatory activity of different brain regions over time, we invited experienced individuals to participate in an EEG recording session with liquid ayahuasca ingestion in a standardized dose in a laboratory setting. To better understand the pharmacological mechanisms related to the effects of ayahuasca on brain rhythms, periodic collection of blood samples was performed. This allowed quantification of the circulating levels of the parent compounds and their metabolites.

## Methods

### Subjects

Twenty healthy volunteers (eight women, mean age, 29.0 years, SD, 3.7 years, mean weight, 55.7 kg, SD, 5.3; 12 men, mean age, 38.5 years, SD, 8.0 years, mean weight, 74.9, SD, 7.7) with previous experience drinking ayahuasca in non-religious settings gave informed consent to participate in the study. All procedures were approved by the Ethical Committee of Universidade Federal de São Paulo and the study was conducted following available guidelines for safety in human hallucinogen research [30]. A psychiatric assessment was conducted, volunteers disclosed their previous drug use histories and signed an informed consent. Exclusion criteria were as follows: < 21 years old, personal history of psychiatric illness, current use of any psychiatric medication, cardiovascular disease and any neurologic disorder or brain injury in the past. Women were asked to participate in the first 15 days of their menstrual cycle. Due to excessive noise in the EEG recording during the baseline period in one experimental session, one male volunteer did not drink ayahuasca and his data were not included in the study.

### Setting

EEG recordings took place in a private standardized room in a psychiatric facility of the Universidade Federal de São Paulo. It was slightly decorated with fabrics on the walls and a plant was put in front of the volunteer's chair. A private bathroom was available with access through a small corridor. During the whole recording each volunteer was accompanied by two researchers (EES and JFMA) and a nurse. Volunteers were never left alone in the room, and they remained seated in a reclining chair. They were asked to remain in "resting-state", i.e. quiet and introspective, with eyes closed. One researcher (EES) was sitting in front of the volunteer, carefully writing down any behavioral manifestation, movements, eye openings as well as following the EEG tracings in a notebook screen. The nurse was sitting by the side and the other researcher in the back of the volunteer's chair. Two buckets were available in case of vomiting (a common effect after ayahuasca ingestion).

### Ayahuasca and plasma samples

A sample of six liters of "Hoasca" was donated by the União do Vegetal (UDV) church in Brazil for the specific purpose of use in this study. Quantification of the main compounds in the brew was done with liquid chromatography-electrospray ionization-tandem mass spectrometry (LC/MS/MS) [6]. The screening included the following compounds: DMT, DMT‐N‐oxide (DMT-NO), N‐methyltryptamine (NMT), indoleacetic acid (IAA), 5‐hydroxy‐DMT (5-OH-DMT, or bufotenin), 5‐methoxy‐DMT (5-MeO-DMT), Harmine, Harmol, Harmaline, Harmalol, THH, 7‐hydroxy‐tetrahydroharmine (THH-OH), and 2‐methyl‐tetrahydro‐beta‐carboline (2-MTHBC). The original liquid volume was then reduced to 50% using a rotary evaporator to decrease the amount of liquid intake.

Blood was collected in sterile EDTA containing tubes. Ten blood samples (3 mL each) were collected every 25 minutes, starting 25 minutes before ayahuasca ingestion and ending 200 minutes after ingestion (in a total of ten samples for each individual). Blood was collected by a professional nurse, with a catheter that remained in place during the entire time of the study. Each tube was carefully and slowly turned upside-down three times and then stored at 0°C until all ten samples were collected. Blood samples were then centrifuged at 2000 rpm for 10 min at 4°C and the resulting plasma immediately collected and frozen at -80°C. The frozen plasma samples were stored at -80°C until shipped on dry ice to USA for analysis according to [31]. Compounds included in this screening were DMT, DMT-NO, NMT, IAA, 5-OH-DMT (bufotenin), 5-MeO-DMT, Harmine, Harmol, Harmaline, Harmalol, THH, THH-OH and 2-MTHBC.

### Subjective effects

Subjective effects were assessed using the Hallucinogen Rating Scale—Brazilian Version [32] and qualitative interviews were done immediately after the end of the EEG recordings.

### Electroencephalography (EEG)

Electroencephalogram recordings were made for two consecutive hours using a Brain Vision ActiCHamp with 64 channels using the standard ActiCap with PyCorder software. Recordings were referenced to Cz and re-referenced offline to the common average. Impedance was considered optimum if < 15 kOhm but still acceptable if > 15 and < 35 kOhm. This was achieved in 80% of the channels. Channels with impedance > 35 kOhm were marked for further offline evaluation. Sampling frequency was 2 kHz and no filters were applied during the recordings. To avoid line noise and other sources of interference, the whole setup (recording equipment and notebook) were connected only to an external battery independently of the power line of the building.

Two subjects had their whole EEG data discarded due to excess movement during the whole recording period. Data from the remaining subjects was divided in ten minute windows immediately preceding each blood sample collection. The first such segment was before drinking ayahuasca (T0, baseline) and the remaining after 25, 50, 75, 100 and 125 min from ingestion. These were preprocessed using EEGLab v.13.3.2b [33]. All recordings were downsampled to 500 Hz and bandpass filtered between 0.5 and 150 Hz with an FIR filter and subsequently visually inspected for identification and removal of bad segments due to movements. Percent of data loss at each time window, from baseline to 125 minutes post ingestion was as follows (mean ± SD): 14.79 ± 11.61, 4.64 ± 2.57, 12.38 ± 13.63, 14.57 ± 15.20, 8.83 ± 7.15 and 8.57 ± 9.66. Bad channels, identified by visual inspection of the tracings and power spectrum, were removed and interpolated using spherical splines [34]. The remaining data were then segmented in 3 s consecutive epochs and all epochs with any discontinuity due to previous removal of bad segments were also excluded. The epoched data of each time window were then processed using the infomax independent component analysis algorithm (implemented as runICA in EEGLab). Components with characteristic of line noise (concentrated spectral peak at 60 Hz), muscle artifacts (elevated spectral power beyond 15–20 Hz and topographic distribution around the edges of the head) or eye blinks and eye movements (spectral peaks at very low frequencies, i.e. 1–2 Hz, and visible slow oscillations in the filtered tracings) were removed from the data. Special attention was given to components with characteristics of muscle artifacts, since was difficult at times for participants to stay still during the effects of ayahuasca.

After preprocessing in EEGLab the data were exported to Fieldtrip [35] for further spectral analysis and statistical procedures. The power spectrum was calculated with a hanning window for frequencies between 1 and 30 Hz and with slepian multitapers for frequencies from 30 to 100 Hz using ± 10 Hz smoothing [36]. The following six frequency bands were defined as of interest for further analysis: delta (1–4 Hz), theta (4–8 Hz), alpha (8–13 Hz), beta (13–30 Hz), slow-gamma (30–50 Hz) and fast-gamma (50–100 Hz).

### Statistical analysis

Spectral changes in each frequency band were evaluated using permuted F statistic (5000 randomizations) corrected for multiple comparisons using a non-parametric cluster-based approach [37]. Post-hoc analysis of significant clusters in the F Statistic was done using Dunnet’s test, comparing each post-ingestion segment with the baseline.

Relationships between plasma concentration of identified compounds and spectral changes in the six frequency bands evaluated were calculated by fitting generalized linear models (glmfit function in MATLAB) for each compound and frequency band. This analysis was followed by the non-parametric cluster based correction with 5000 permutations [37].

Results were considered statistically significant when p <0.05. Effect sizes are also reported, using Partial eta squared (partial η^2^) for the main frequency clusters and Cohen’s *d* for the generalized linear models.

## Results

### Previous experience with ayahuasca and other psychoactive substances

The patients’ lifetime experience with ayahuasca and psychoactive substances, especially psychedelics, varied greatly. First ayahuasca experience happened 7.5 ± 3.6 years (mean ± SD) before the study. The estimated mean number of experiences was 96.7 (SD 135.2), with the minimum being 5 and the maximum 500. Number of experiences in the past year was 10.0 ± 8.0 (mean ± SD), with the range between 0 and 30. In the past month it was 1.3 1.4 (mean ± SD) with the range being 0–5. Curiosity was the most frequent reason for the first ayahuasca use (53%). Other reasons mentioned to start drinking ayahuasca included self-knowledge, contact with the divine, going with friends and consciousness expansion. All volunteers reported getting benefits from regular ayahuasca use. These included healing body and mind, self-knowledge, fear and anxiety reduction, improvement of personal relationships, more contact with nature and cultivating compassion. Three volunteers were uncomfortable with the prospect of blood sample collection (two opted out of this procedure but still participated in the EEG study). One volunteer referred to anxiety with the urban environment and small room, which he did not consider appropriate for an ayahuasca experience, and one volunteer referred to anxiety about the possible anxiogenic effects of street traffic noise negatively interfering in the experience.

Previous experience with other psychoactive substances included tobacco (in form of industrial cigarettes, organic tobacco or indigenous “rapé”, 100%), alcohol (100%), cannabis (82%), psychedelics (LSD, San Pedro or Wachuma, mushrooms, 82%), ecstasy (65%), cocaine (59%), tranquilizers (29%), amphetamines (24%), anticholinergics (18%), opiates (18%).

### Ayahuasca composition

The concentration of the main active ingredients in the ayahuasca used in this study was (in mg/ml): 0.328 (DMT), 1.08 (Harmine), 0.18 (Harmaline) and 1.28 (THH) as shown in Table 1. The dose (in mg/kg) was 1.39 for DMT, 4.58 for Harmine, 0.75 for Harmaline and 5.43 for THH. Concentration of other identified compounds in the tea, respective administered doses and total amount ingested are summarized in Table 1. The following compounds were not detected in the ayahuasca sample: 5-MeO-DMT, 2-MTHBC, DMT-NO and IAA.

**Table 1:** Concentration of chemical compounds in Ayahuasca.

Total mg ingested (min—max)	Total mg ingested (mean ± sd)	Estimated Dose	*Hoasca* Concentration	Compound
64.67–114.73	91.13 ± 14.81	1.39	0.328	**DMT**
3.35–5.95	4.72 ± 0.77	0.07	0.017	**NMT**
-	-	-	ND	**DMT-NO**
0.39–0.70	0.56 ± 0.09	0.01	0.002	**5-OH-DMT**
-	-	-	ND	**5-MeO-DMT**
212.93–377.78	300.06 ± 48.76	4.58	1.080	**Harmine**
104.10–184.69	146.70 ± 23.84	2.24	0.528	**Harmol**
34.70–61.56	48.90 ± 7.95	0.75	0.176	**Harmaline**
2.37–4.20	3.33 ± 0.54	0.05	0.012	**Harmalol**
252.36–447.74	355.63 ± 57.79	5.43	1.280	**THH**
20.50–36.38	28.90 ± 4.70	0.44	0.104	**THH-OH**
-	-	-	ND	**2-MTHBC**
-	-	-	ND	**IAA**

Concentration (mg/ml) of 13 compounds screened in the *Hoasca* tea, estimated ingested dose (mg/kg) and total amount ingested of each compound (mg).

### Blood levels

**Fig 1:**
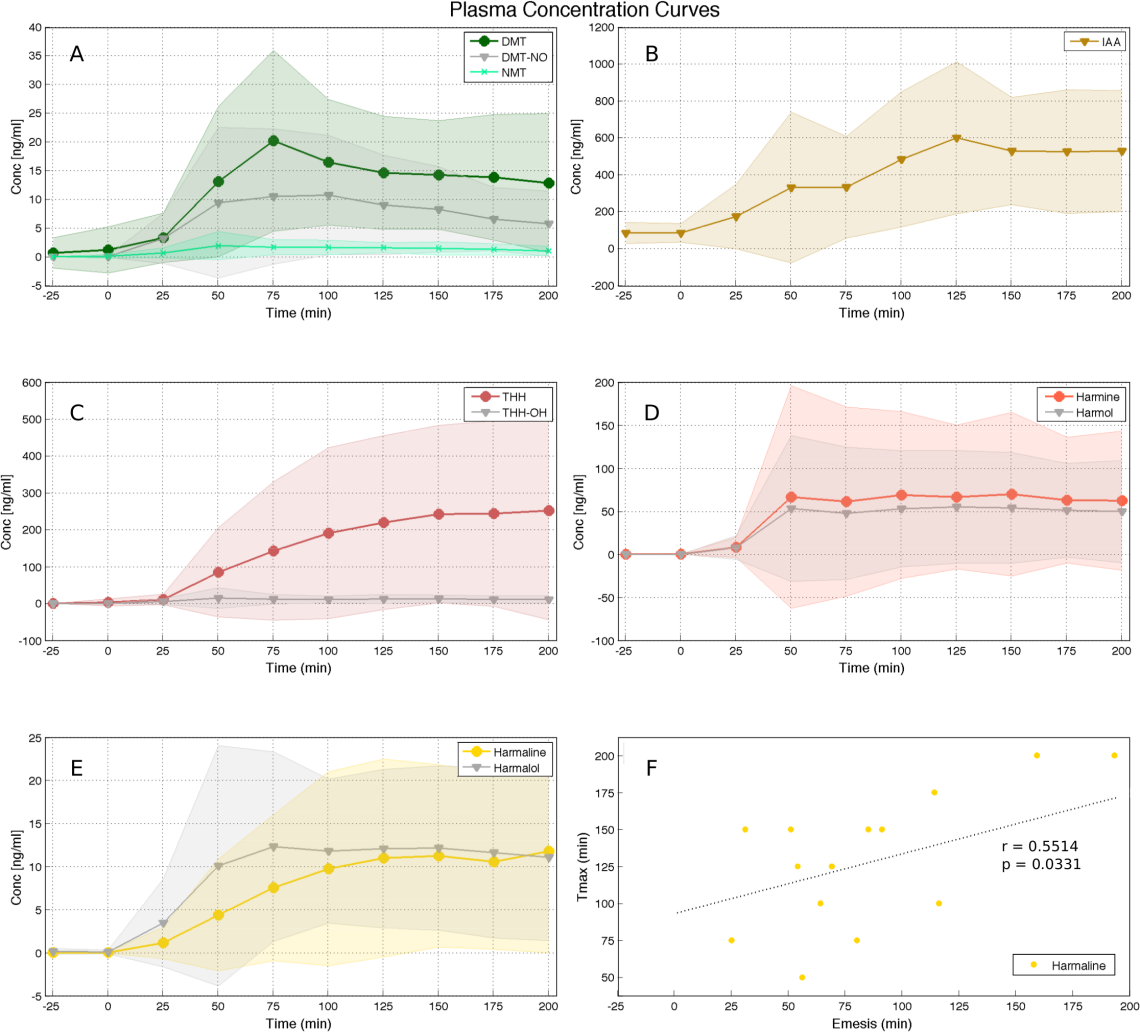
Plasma data. Time-concentration curves for DMT, NMT and DMT-NO (A), IAA (B), THH and THH-OH (C), harmine and harmol (D), harmaline and harmalol (E) and correlation between hamaline Tmax with time for emesis. All concentration data is expressed as mean ± SD in ng/ml. All times expressed in minutes.

Two volunteers opted out of blood collection, and data from one volunteer was discarded due to high hemolysis in the blood samples. Therefore data were available from 16 volunteers. Maximum plasma Concentration (Cmax), Time for maximum plasma concentration (Tmax) and area under the curve (AUC) of ten identified compounds are summarized in Table 2. The following compounds were never detected in any sample: 2-MTHBC, 5-OH-DMT, or 5-MeO-DMT. THH-OH was never detected in two subjects and detected in one out of ten samples of another volunteer. Harmol was never detected in two other subjects. Time-concentration curves are shown in Fig 1. No significant differences were found between men and women for any of the identified compounds in blood. Correlations between concentrations in blood and age were significant only for IAA, where a positive correlation was found between AUC and age (R^2^ = 0.3516, p = 0.0439) and also between Cmax and age (R^2^ = 0.3392, p = 0.0179). Positive correlations between DMT AUC and age, and Cmax and age, were marginally significant (R^2^ = 0.2044, p = 0.0787 and R^2^ = 0.1899, p = 0.0915, respectively). All but one of the participants vomited (95.33 61.07 min, range 25–242). There were no significant correlations between time to vomit and Cmax or AUC. A positive correlation between Tmax and time to vomit was significant for harmaline (R^2^ = 0.2783, p = 0.0433, Fig 1F) and marginally significant for THH (R^2^ = 0.1966, p = 0.0978) and IAA (R^2^ = 0.2256, p = 0.0736).

**Table 2:** Concentration of chemical compounds in plasma samples.

AUC (min—max)	AUC (mean ± sd)	Tmax (mean ± sd)	Cmax (min—max)	Cmax (mean ± sd)	Compound
9.52–219.10	103.42 ± 69.33	132.81 ± 49.77	2.49–65.93	25.39 ± 16.78	**DMT**
1.52–21.69	10.70 ± 6.16	121.88 ± 47.32	0.57–7.56	3.10 ± 2.12	**NMT**
5.21–191.29	60.37 ± 57.60	110.94 ± 37.60	1.58–40.60	14.42 ± 12.50	**DMT-NO**
21.37–2325.28	191.29 ± 580.10	150.00 ± 52.44	5.93–511–54	110.26 ± 137.85	**Harmine**
0.00–1550.35	345.88 ± 407.46	148.21 ± 55.00	0.00–318.79	84.99 ± 93.57	**Harmol**
6.16–199.78	61.39 ± 60.47	160.94 ± 46.52	1.56–44.37	14.68 ± 13.93	**Harmaline**
9.91–204.99	79.04 ± 63.51	125.00 ± 38.73	3.11–48.55	17.22 ± 13.64	**Harmalol**
75.01–3762.73	1262.09 ± 1270.39	190.63 ± 42.70	22.62–895.70	328.76 ± 324.86	**THH**
0.00–284.32	3762.73 ± 81.29	119.64 ± 53.87	0.00–111.01	22.58 ± 26.76	**THH-OH**
1237.81–7726.25	284.32 ± 2077.85	175.00 ± 39.79	61.35–1674.57	717.72 ± 454.50	**IAA**

Values obtained for 10 compounds detected in plasma samples. Cmax values are expressed in ng/ml, Tmax values in min, AUC in g min/ml.

### Subjective effects

**Fig 2:**
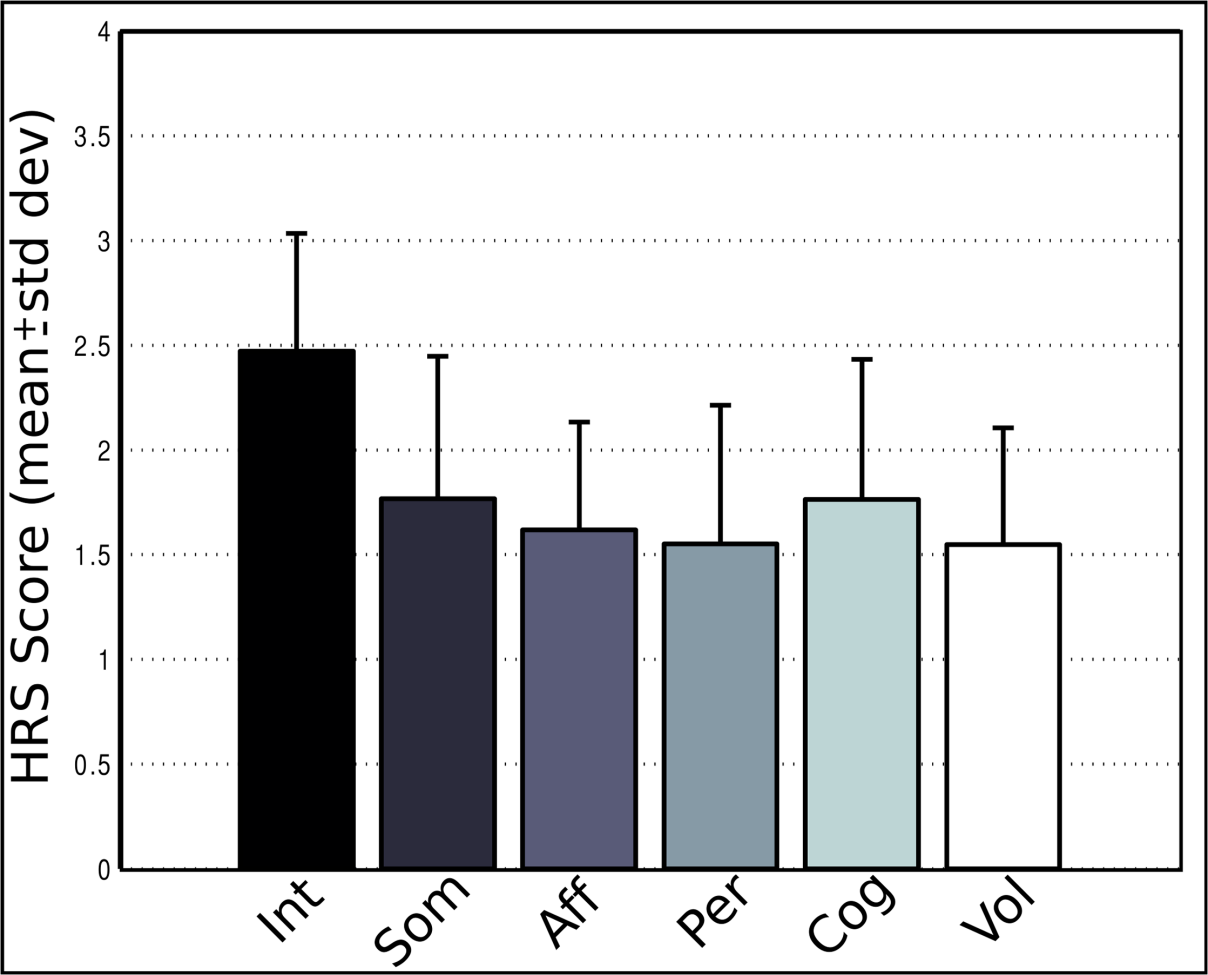
HRS subjective ratings. Subjective results as measured with the Hallucinogen Rating Scale–Brazilian Version. Int = Intensity, Som = Somaesthesia, Aff = Affect, Per = Perception, Cog = Cognition and Vol = Volition. For all six sub scales four is the maximum score possible.

All participants reported noticeable changes in their normal state of consciousness after ingesting ayahuasca. The most common effects included visual perceptions with eyes closed, heightened sensitivity to ambient noises (especially street traffic), alterations in perception of time and space, increased imagination, introspection and emotional arousal. Fig 2 shows subjective ratings obtained with HRS—Brazilian Version.

### Spectral Analysis

**Fig 3:**
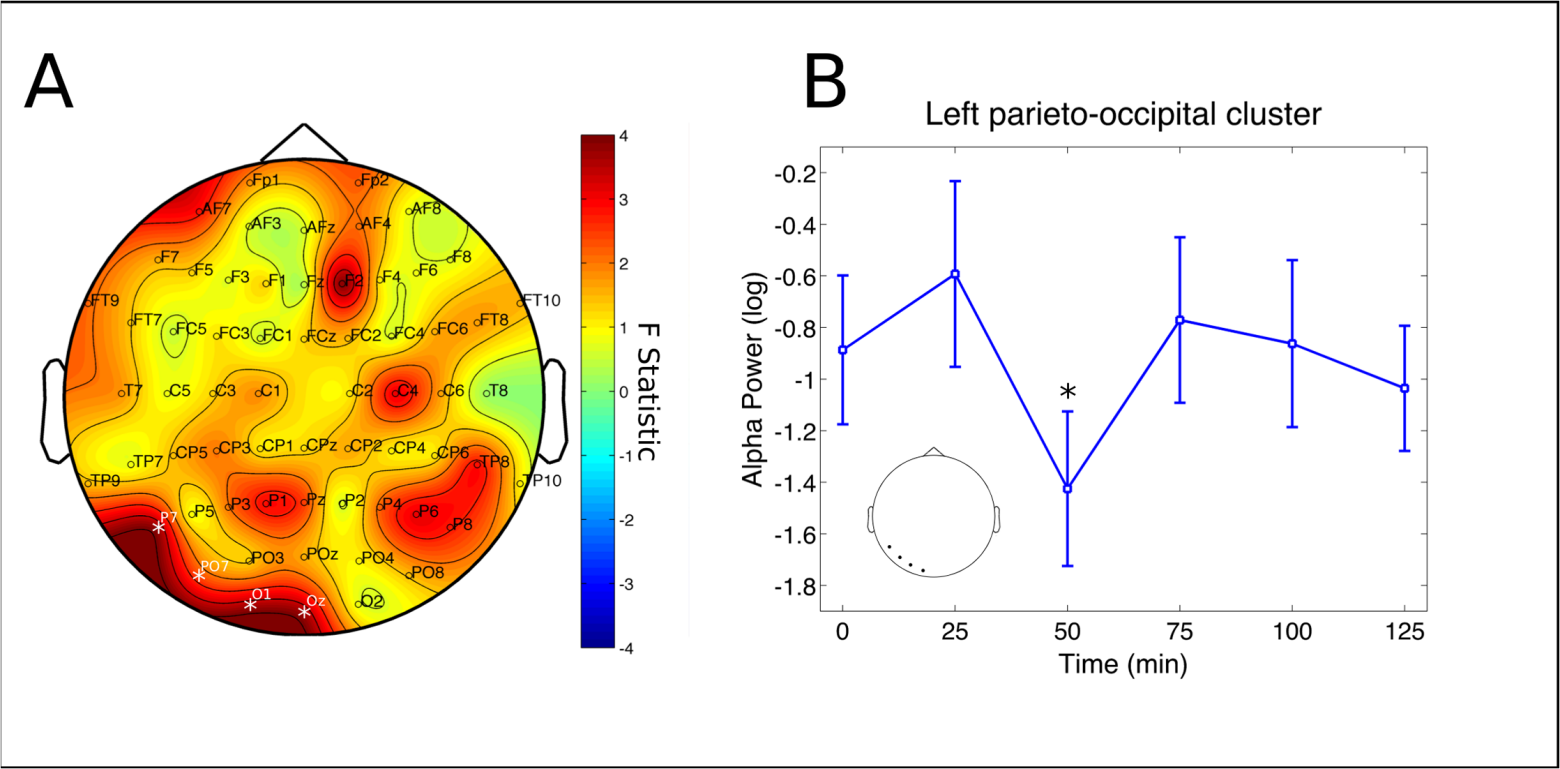
Alpha rhythm cluster. Changes in alpha frequency band power spectrum over the 2 hours since ayahuasca ingestion. A) F statistics in the entire scalp revealed a significant cluster at left parieto-occipital electrodes (P7, PO7, O1 and Oz, p = 0.0398, highlighted by white *). B) Post-hoc analysis revealed decreases in the cluster depicted at left to be significant after 50 minutes from ingestion of ayahuasca (*p<0.05, corrected). Inset highlights the electrodes averaged for the post-hoc analysis, in correspondence with A. Data in B is expressed as mean and standard error.

**Fig 4:**
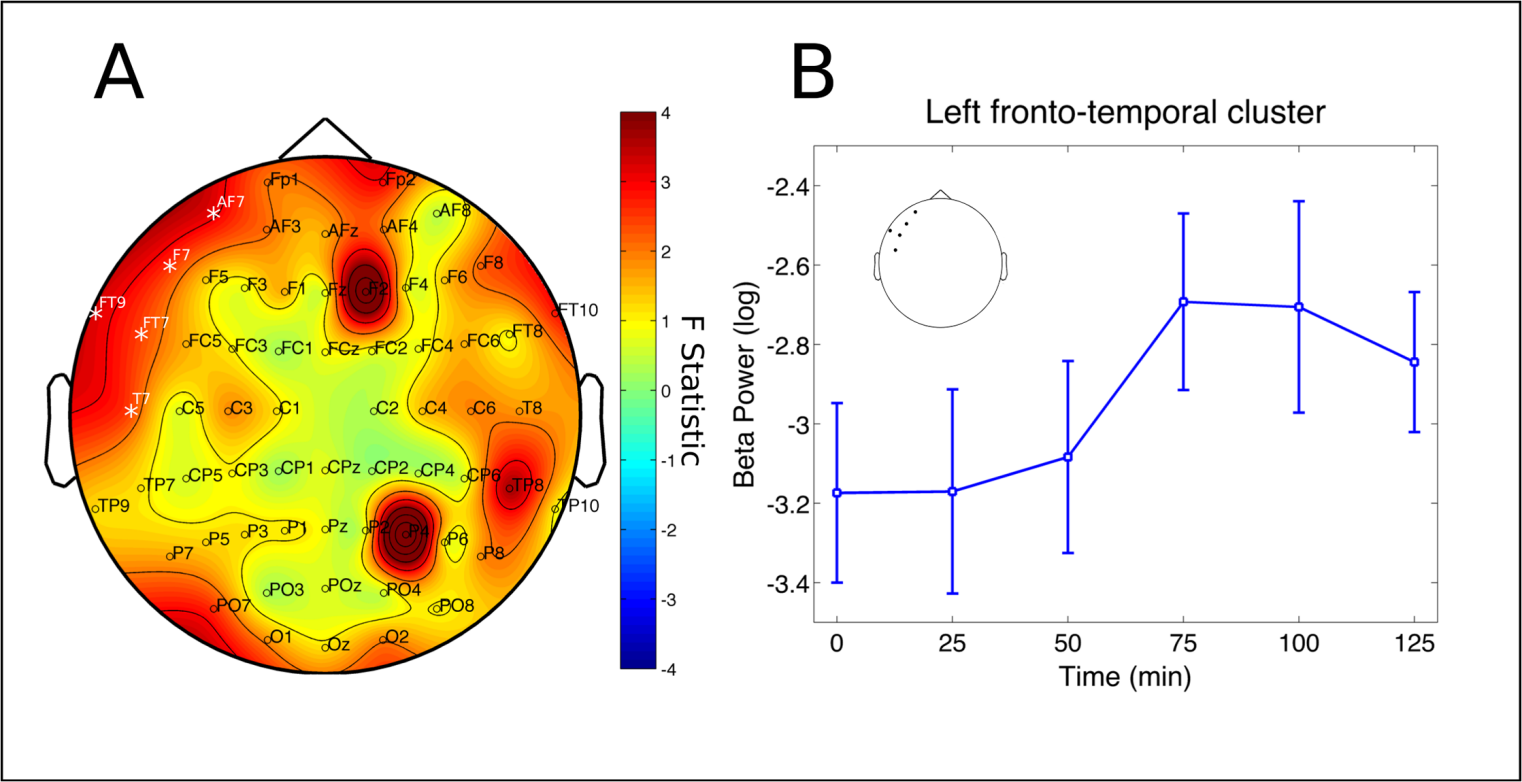
Beta rhythm cluster. Changes in beta frequency band power spectrum over the 2 hours since ayahuasca ingestion. A) F statistics in the entire scalp revealed a significant cluster at left fronto-temporal electrodes (FT9, AF7, F7, FT7 and T7, p = 0.0260, highlighted by white *). B) Beta band increases did not survive post-hoc analysis. Inset highlights the electrodes averaged for the post-hoc analysis, in correspondence with A. Data in B is expressed as mean and standard error.

**Fig 5:**
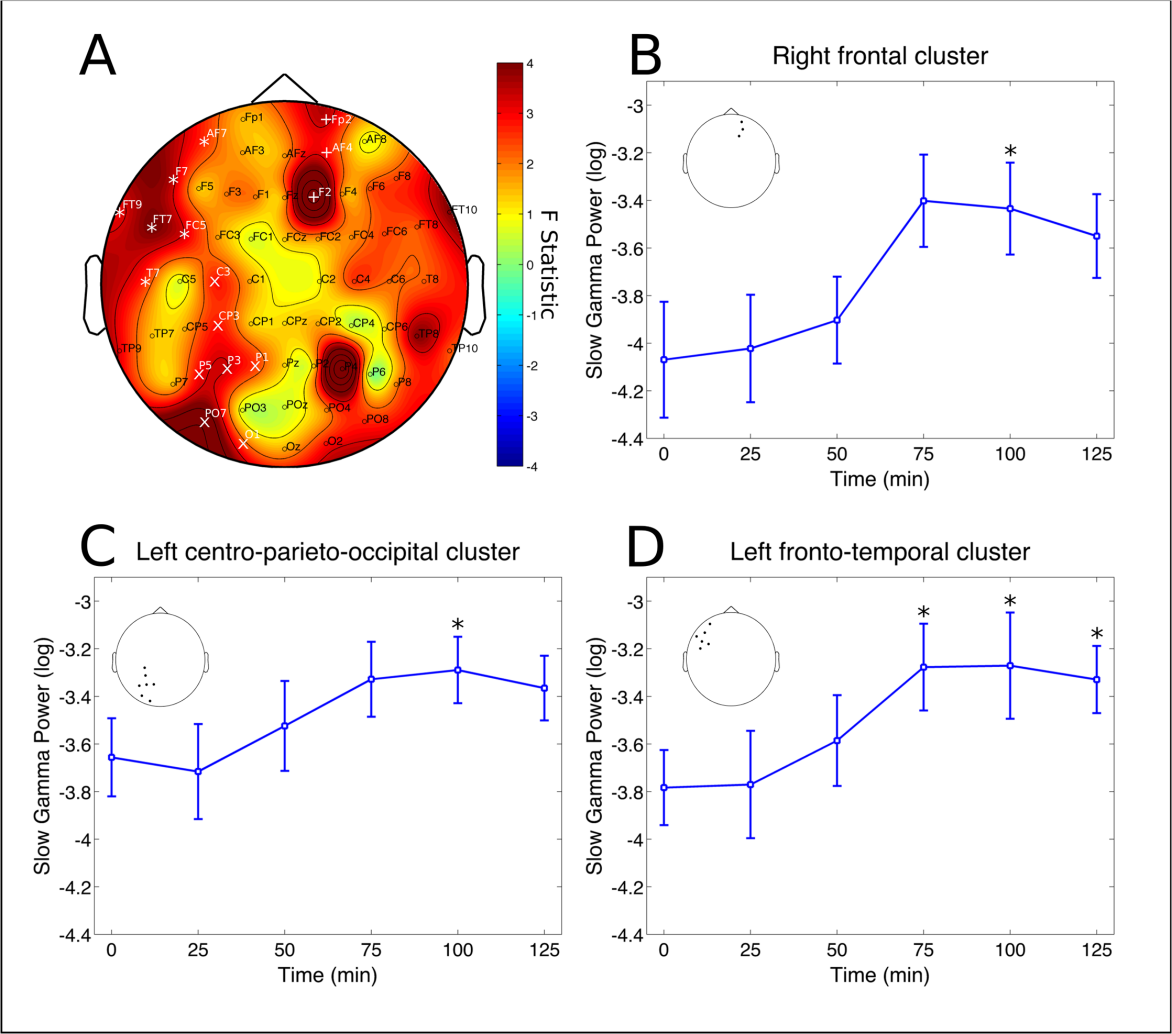
Slow-gamma rhythm clusters. Changes in slow-gamma frequency band power spectrum over the 2 hours since ayahuasca ingestion. A) F statistics in the entire scalp revealed three significant clusters at the right frontal (Fp2, AF4 and F2, p = 0.0046, highlighted by white *), left centro-parieto-occipital (C3,CP3, P5, P3, P1, PO7 and O1, p = 0.0152, highlighted by white x) and left fronto-temporal (FT9, AF7, F7, FT7, T7 and C5, p = 0.0026, highlighted by white +). B) Slow-gamma band power increases at right frontal cluster were significant only after 100 min from ayahuasca ingestion (*p<0.05, corrected). C) Slow-gamma band power increases at left centro-parieto-occipital cluster were significant only after 100 min from ayahuasca ingestion (*p<0.05, corrected). D) Slow-gamma band power increases at left fronto-temporal cluster were significant after 75, 100 and 125 min from ayahuasca ingestion (*p<0.05, corrected). Insets in B, C and D highlight the electrodes averaged for each post-hoc analysis, in correspondence with A. Data in B, C and D is expressed as mean and standard error.

**Fig 6:**
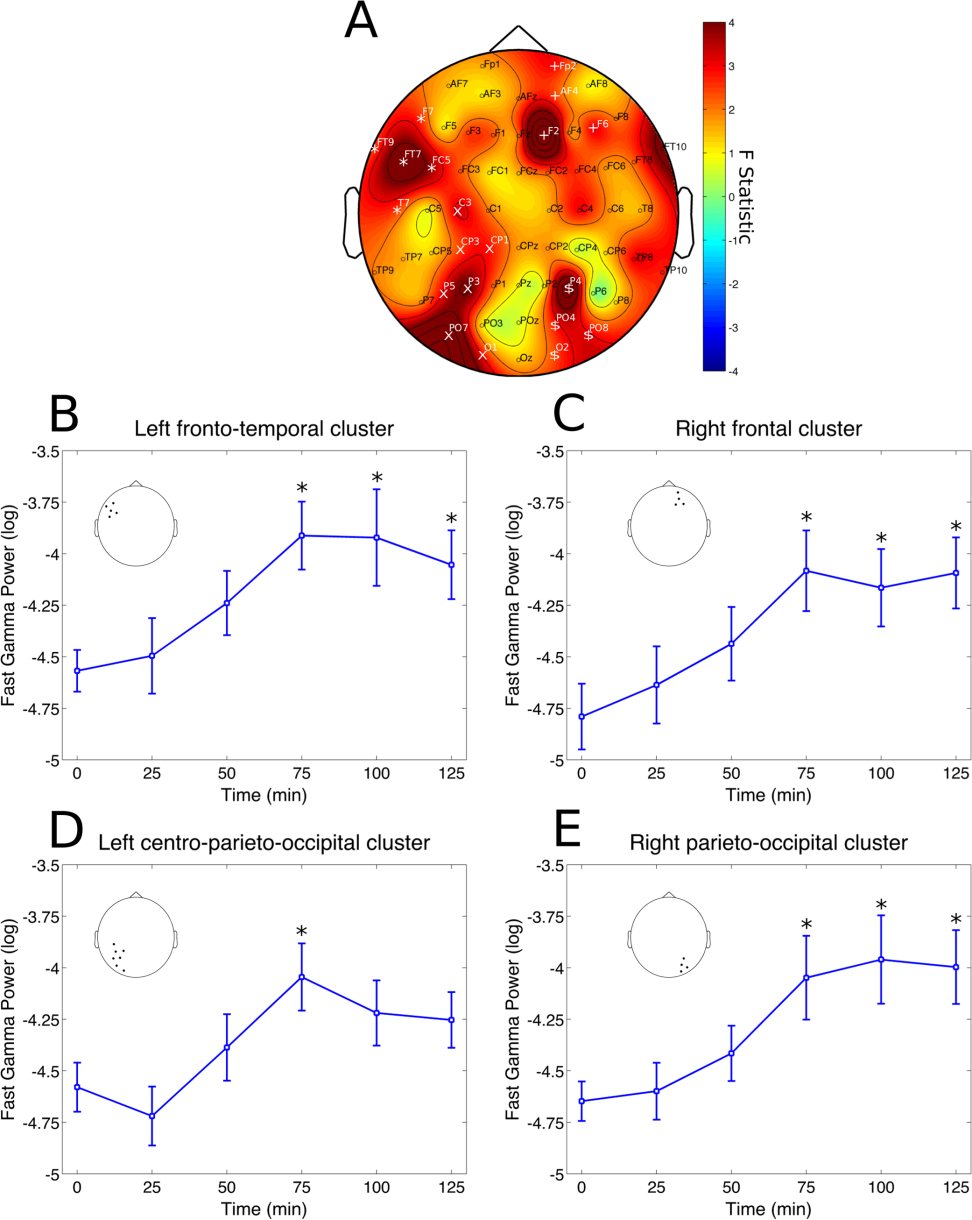
Fast-gamma rhythm clusters. Changes in fast-gamma frequency band power spectrum over the 2 hours since ayahuasca ingestion. A) F statistics in the entire scalp revealed four significant clusters at right-frontal (Fp2, AF4, F2 and F6, p = 0.0016, highlighted by white +), right parieto-occipital (P4, PO4, O2 and PO8, p = 0.0088, highlighted by white $), left fronto-temporal (FT9, F7, FT7, FC5 and T7, p = 0.0098, highlighted by white *) and left centro-parieto-occipital region (C3, CP3, CP1, P5, P3, PO7 and O1, p = 0.0132, highlighted by white x). B) Fast-gamma band power increases at left fronto-temporal cluster were significant after 75, 100 and 125 min from ayahuasca ingestion (*p<0.05, corrected). C) Fast-gamma band power increases at right frontal cluster were significant after 75, 100 and 125 min from ayahuasca ingestion (*p<0.05, corrected). D) Fast-gamma band power increases at left centro-parieto-occipital cluster were significant only after 75 min from ayahuasca ingestion (*p<0.05, corrected). E) Fast-gamma band power increases at right parieto-occipital cluster were significant after 75, 100 and 125 min from ayahuasca ingestion (*p<0.05, corrected). Insets in B, C, D and E highlight the electrodes averaged for each post-hoc analysis, in correspondence with A. Data in B, C, D and E is expressed as mean and standard error.

No significant changes were found in the delta and theta bands. A cluster at left parieto-occipital electrodes (P7, PO7, O1 and O2) had significant decreases in alpha power (p = 0.0398, partial η^2^ = 0.1531, Fig 3A). Post-hoc analysis revealed this effect to be significant specifically after 50 min from ayahuasca ingestion (p = 0.0420 Fig 3B). A significant cluster was also found for the beta band, at left fronto-temporal electrodes (FT9, AF7, F7, FT7 and T7, p = 0.0260, partial η^2^ = 0.1946, Fig 4A). However, this effect did not survive statistical thresholds after post-hoc analysis (Fig 4B). In the slow-gamma band three different clusters were found in the right frontal (Fp2, AF4 and F2, p = 0.0046, partial η^2^ = 0.3067), left fronto-temporal (FT9, AF7, F7, FT7, T7 and C5, p = 0.0026, partial η^2^ = 0.2214) and left centro-parieto-occipital (C3,CP3, P5, P3, P1, PO7 and O1, p = 0.0152, partial η^2^ = 0.2555) (Fig 5A). Slow-gamma power in these three clusters increased specifically after 100 minutes from ingestion of ayahuasca, with exception of the left fronto-temporal cluster where the increase was also significant at 75 and 125 min post-ingestion (all ps <0.05, Fig 5B, 5C and 5D, respectively). In the fast-gamma range four different clusters were identified. These were located at right-frontal (Fp2, AF4, F2 and F6, p = 0.0016, partial η^2^ = 0.3076), right parieto-occipital (P4, PO4, O2 and PO8, p = 0.0088, partial η^2^ = 0.2850), left fronto-temporal (FT9, F7, FT7, FC5 and T7, p = 0.0098, partial η^2^ = 0.2506) and left centro-parieto-occipital region (C3, CP3, CP1, P5, P3, PO7 and O1, p = 0.0132, partial η^2^ = 0.2870) (Fig 6A). Post-hoc analysis revealed these increase to be significant at 75, 100 and 125 min post-ayahuasca ingestion, with the exception of the left centro-parieto-occipital cluster where it was significant only after 75 min (all ps < 0.05, Fig 6B, 6C, 6D and 6E).

### Plasma concentration and EEG spectra

**Fig 7:**
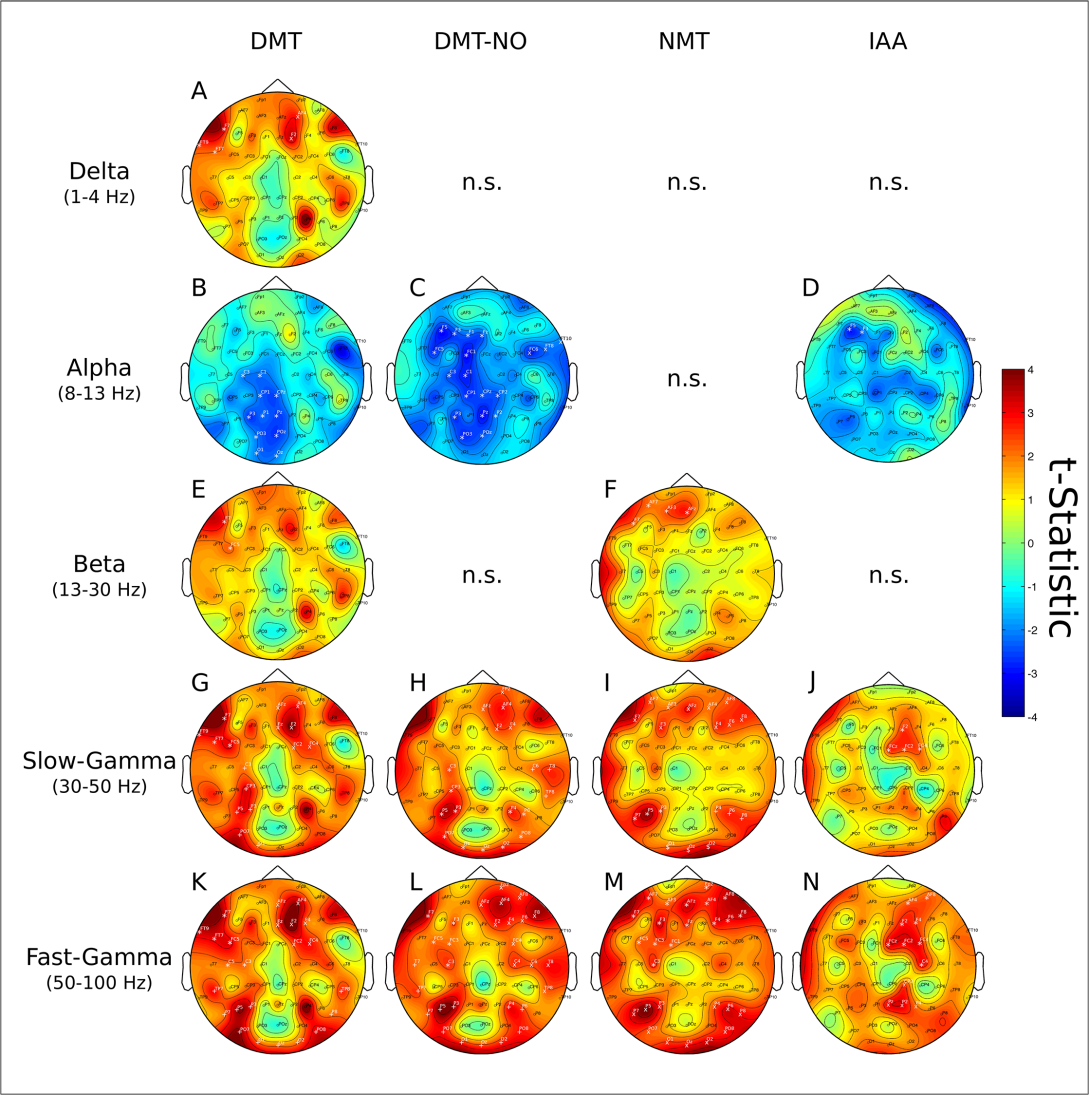
Tryptamine’s concentration and EEG effects. Statistical associations between plasma levels of DMT (first column from left), DMT-NO (second column), NMT (third column) and IAA (fourth column) with the delta, alpha, beta, slow- and fast-gamma frequency bands. No significant effects were found with the theta band. A) DMT and delta frequency band. B) DMT and alpha frequency band. C) DMT-NO and alpha frequency band. D) IAA and alpha frequency band. E) DMT and beta frequency band. F) NMT and beta frequency band. G) DMT and slow-gamma frequency band. H) DMT-NO and slow-gamma frequency band. I) NMT and slow-gamma frequency band. J) IAA and slow-gamma frequency band. K) DMT and fast-gamma frequency band. L) DMT-NO and fast-gamma frequency band. M) NMT and fast-gamma frequency band. N) IAA and fast-gamma frequency band. Corresponding p values and Cohen’s *d* for each cluster are shown in the main text.

**Fig 8:**
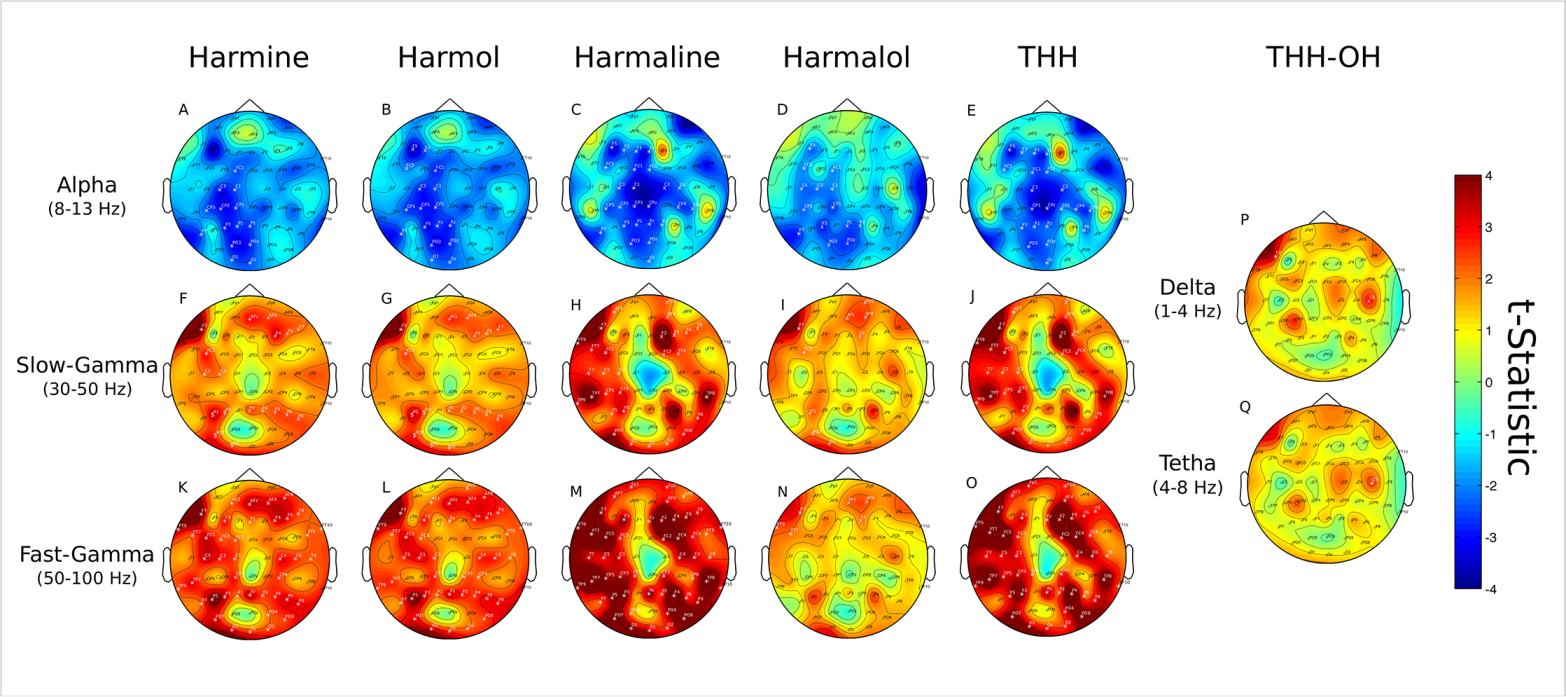
Beta-carboline’s concentration and EEG effects. Statistical associations between plasma levels of the beta-carbolines harmine (first column from left), harmol (second column), harmaline (third column), harmalol (fourth column), THH (fifth column) and THH-OH (last column) with delta, theta (only for THH-OH) and alpha, slow- and fast-gamma frequency bands (not significant for THH-OH). A) Harmine and alpha. B) Harmol and alpha. C) Harmaline and alpha. D) Harmalol and alpha. E) THH and alpha. F) Harmine and slow-gamma. G) Harmol and slow-gamma. H) Harmaline and slow-gamma. I) Harmalol and slow-gamma. J) THH and slow-gamma. K) Harmine and fast-gamma. L) Harmol and fast-gamma. M) Harmaline and fast-gamma. N) Harmalol and fast-gamma. O) THH and fast-gamma. P) THH-OH and delta. Q) THH-OH and theta. Corresponding p values and Cohen’s *d* for each cluster are shown in the main text.

Associations between EEG power spectral changes and plasma concentration curves of the tryptamines DMT, DMT-NO, NMT and IAA are shown in Fig 7. Results for the beta-carbolines harmine, harmol, harmaline, harmalol, THH and THH-OH with each frequency band are shown in Fig 8.

Concentration of DMT and increases in the delta band formed two significant clusters. One in the left fronto-temporal electrodes (FT9, F7 and FT7, p = 0.0044, *d* = 0.2962) and another at right frontal electrodes (AF4 and F2, p = 0.0181, *d* = 0.2100) (Fig 7A). These were not significant for DMT-NO, NMT and IAA.

No significant effects were found between the tryptamines and the theta frequency band. There was one large significant cluster between DMT and alpha band power decrease at left centro-parieto-occipital electrodes (C3, C1, CP1, CPz, P3, P1, Pz, PO3, POz, O1 and Oz, p = 0.0002, *d* = 0.0822, Fig 7B). A similar cluster was found for DMT-NO and alpha band decrease in power at left centro-parieto-occipital and extending further to some left frontal electrodes (F5, F3, F1, Fz, FC5, FC1, C3, C1, CP1, CPz, CP2, P3, Pz, P2, PO3 and POz, p = 0.0002, *d* = 0.0610, Fig 7C). In addition, a localized significant cluster between DMT-NO and alpha power decrease was present at right fronto-temporal electrodes (FC6, FT8 and FT10, p = 0.0340, *d* = 0.1341, Fig 7C). No significant clusters were found for NMT and alpha band. One significant small cluster was found for IAA and alpha band in left frontal electrodes (F5 and F3, p = 0.0320, *d* = 0.2888, Fig 7D).

Localized small clusters were found between DMT and an increase in beta power at left fronto-central electrodes (F7 and FC5, p = 0.0432, *d* = 0.1131, Fig 7E) and between NMT and beta power increase at left frontal electrodes (F7, AF7, AF3, AFz, p = 0.0022, *d* = 0.0863, Fig 7F). No significant clusters were found between DMT-NO and IAA with the beta band.

Three significant clusters were found between slow-gamma power increases and DMT plasma concentration. These were located at left fronto-temporal electrodes (FT9, F7, FT7 and FC5, p = 0.0002, *d* = 0.0966), left centro-parieto-occipital electrodes (C3, CP3, P5, P3, PO7 and O1, p = 0.0002, *d* = 0.0125) and also right frontal electrodes (AFz, AF4, Fz, F2, FC2 and FC4, p = 0.0002, *d* = 0.0161) (Fig 7G). Three clusters were found between DMT-NO and increases in slow-gamma power. These were located at right frontal electrodes (FP2, AFz, AF4, F2, F4, p = 0.0003, *d* = 0.0061), left centro-parieto-occipital extending to right parietal electrodes (C3, CP3, P5, P3, PO7, O1, Oz, O2, PO8, P6 and P4, p = 0.0002, *d* = 0.0593) and right centro-temporal electrodes (C6, T8 and TP8, p = 0.0210, *d* = 0.0052) (Fig 7H). Four significant clusters were found between NMT and slow-gamma increases. One at bilateral frontal electrodes (AF7, AF3, F7, F3, AFz, AF4, AF8, F4, F6 and F8, p = 0.0134, *d* = 0.0822), one at left parieto electrodes (P7, P5 and P3, p = 0.0074, *d* =), one at right parietal electrodes (P4, P6 and P8, p = 0.0026, *d* = 0.1538) and one at occipital electrodes (O1, Oz and O2, p = 0.0002, *d* = 0.2579) (Fig 7I). One significant cluster was found between IAA and slow-gamma at right centro-frontal electrodes (F2, FCz, FC2, FC4, p = 0.0014, *d* = 0.0052, Fig 7J).

Fast-gamma power increases and DMT concentration yielded three significant clusters at left fronto-temporal electrodes (FT9, F7, FT7, FC5, p = 0.0002, *d* = 0.0193), right-frontal electrodes (AFz, AF4, Fz, F2, F4, FC2, FC4, p = 0.0002, *d* = 0.1366) and at bilateral temporo-parieto-occipital electrodes (TP7, P7, P5, P3, PO7, O1, Oz, O2, PO8, P8, TP8, p = 0.0002, *d* = 0.0309) (Fig 7K). Three significant clusters were found between fast-gamma increases and DMT-NO concentrations. These were located at left centro-frontal electrodes (F7, F3, FC5, FC3 and C3, p = 0.0004, *d* = 0.1917), right centro-temporo-frontal electrodes (Fp2, AFz, AF4, AF8, F2, F4, F6, F8, FC4, C4, C6, T8 and TP8, p = 0.0002, *d* = 0.0269) and at bilateral parieto-occipital electrodes (P7, P5, P3, PO7, O1, Oz, O2, PO8, P6, P4, p = 0.0002, *d* = 0.0003) (Fig 7L). Fast-gamma power increases and NMT concentration revealed two significant large clusters. One at bilateral fronto-central electrodes (AF7, AF3, F7, F3, FC5, FC3, FC1, C3, AFz, Fz, Fp2, AF4, AF8, F4, F6 and F8, p = 0.0002, *d* = 0.0290) and the other at bilateral parieto-occipital electrodes (P7, P5, P3, PO7, O1, Oz, O2, PO8, P4, P6 and P8, p = 0.0002, *d* = 0.0911) (Fig 7M). Two significant clusters were found between IAA and fast-gamma. One at right frontal electrodes (AF4, AF8, F2, F4, FCz, FC4, C4, p = 0.0002, *d* = 0.0737) and the other at right parietal electrodes (CP2, Pz, P2, P4, p = 0.0034, *d* = 0.1428, Fig 7N).

Concentrations of harmine, harmol, harmaline, harmalol and THH with EEG spectra did not show any significant clusters in the delta, theta and beta frequency bands. Alpha band power decreases and harmine concentration formed one significant cluster at left centro-parieto-occipital electrodes (FC1, C3, C1, CP5, CP3, CP1, P7, P3, P1, Pz, PO3, POz, O1 and Oz, p = 0.0002, *d* = 0.0297, Fig 8A). Alpha band power decrease and harmol concentration formed a similar cluster at left centro-parieto-occipital electrodes (FC1, C3, C1, CP5, CP3, CP1, P7, P3, P1, Pz, PO3, POz, O1 and Oz, p = 0.0002, *d* = 0.0556) and also a small cluster at left frontal electrodes (F5, F3 and FC5, p = 0.0460, *d* = 0.3029) (Fig 8B). Power decreases in the alpha band and harmaline concentration formed two significant clusters at left centro-parieto-occipito-frontal electrodes (F5, F3, F1, Fz, FC5, FC1, FCz, C3, C1, CP5, CP3, CP1, CPz, P7, P3, P1, Pz, P2, PO3, POz and Oz, p = 0.0002, *d* = 0.0285) and right central electrodes (C2, C4, CP4 and CP6, p = 0.0026, *d* = 0.2944) (Fig 8C). Harmalol concentration and alpha band power decrease formed two significant clusters at left centro-parietal (F5, FC5, FC1, C5, C3, C1, CP5, CP1 and P7, p = 0.0002, *d* = 0.0673) and left parieto-occipital electrodes (P3, Pz, P2, PO3 and POz, p = 0.0002, *d* = 0.1056) (Fig 8D). Alpha power decrease and THH concentration formed one significant cluster at left fronto-centro-parieto-occipital cluster extending also to some right centro-parietal electrodes (F5, F3, F1, Fz, FC5, FC1, C1, CP1, P3, P1, PO3, Fz, FCz, CPz, Pz, POz, Oz, C2, CP2, CP4, CP6 and P6, p = 0.0002, *d* = 0.0044, Fig 8E). No significant clusters were found for the alpha band and THH-OH concentration.

There were four significant clusters between slow-gamma power increases and harmine concentration. These were at left centro-frontal electrodes (F7, FC5, C5 and C3, p = 0.0010, *d* = 0.2028), right frontal electrodes (AFz, AF4, F4, F6 and F7, p = 0.0020, *d* = 0.0024), left parieto-occipital electrodes (P5, P3, PO7 and O1, p = 0.0022, *d* = 0.1797) and right parietal electrodes (Pz, P2, P4, P6 and P8, p = 0.0036, *d* = 0.1208) (Fig 8F). Slow-gamma power increases and harmol concentrations also formed four significant clusters. One at left fronto-central electrodes (F7 and FC5, p = 0.0010, *d* = 0.1250), one at right frontal electrodes (AFz, AF4, F4, F6 and F7, p = 0.0020, *d* = 0.2747), one at left parieto-occipital electrodes (P5, P3, PO7, O1 and Oz, p = 0.0024, *d* = 0.2921) and one at right parietal electrodes (Pz, P2, P4, P6 and P8, p = 0.0452, *d* = 0.0941) (Fig 8G). Power increase in the slow-gamma frequency band and harmaline concentration formed two significant clusters. One at right frontal electrodes (Fp2, AF4, F2, F4, F6, F8, FC2 and FC4, p = 0.0002, *d* = 0.0401) and the other at left fronto-temporo-centro-parieto-occipital electrodes also extending to right temporo-parietal electrodes (AF7, F7, FT9, FT7, FC5, T7, C5, C3, TP9, TP7, CP5, CP3, P5, P3, PO7, O1, Oz, O2, PO8, P8 and TP8, p = 0.0002, *d* = 0.0885) (Fig 8H). Slow-gamma power increase and harmalol concentration formed three localized clusters at left fronto-central electrodes (F7 and FC5, p = 0.0002, *d* = 0.0707), right frontal electrodes (Fp2, AFz, AF4 and F2, p = 0.0020, *d* = 0.001) and left parieto-occipital electrodes (P5, PO7 and O1, p = 0.0058, *d* = 0.0542) (Fig 8I). Slow-gamma power increase and THH concentration formed three significant clusters. These were located at right frontal electrodes (Fp2, AF4, F2, F4, FC2 and FC4, p = 0.0002, *d* = 0.0366), left fronto-temporo-central electrodes (AF7, F7, FT9, FT7, FC5, T7, C5, C3, TP9, TP7, CP5, CP3 and P3, p = 0.0002, *d* = 0.0795) and a bilateral parieto-occipital cluster (PO7, O1, Oz, O2, PO8, P8 and TP8, p = 0.0002, *d* = 0.0656) (Fig 8J). No significant clusters were found for THH-OH and slow-gamma frequency band.

Fast-gamma power increases and harmine concentration were widespread, forming one large bilateral cluster (AF3, F7, F3, FT9, FT7, FC5, FC3, FC1, AFz, Fz, AF4, AF8, F4, F6, F8, FT6, FT10, C5, C3, C1, C4, C6, T8, TP9, TP7, CP1, CP4, P7, P5, P3, P1, Pz, P2, P4, P6, P8, PO7, O1, Oz, O2, PO4 and PO8, p = 0.0002, *d* = 0.0312, Fig 8K). A similar widespread cluster was found between harmol concentration and fast-gamma power increase (AF3, F7, F3, FT9, FT7, FC5, FC3, C5, C3, C1, AFz, Fz, Fp2, AF4, AF8, F4, F6, F8, FT6, FT10, C4, C6, T8, TP9, TP7, CP1, CP2, CP4, P7, P5, P3, P1, Pz, P2, P4, P6, P8, PO7, O1, Oz, O2 and PO8, p = 0.0002, *d* = 0.0463, Fig 8L). Fast-gamma power increase and harmaline concentration resulted in one widespread cluster reaching electrodes in all regions bilaterally (Fp1, AF7, F7, F3, FT9, FT7, FC5, FC3, T7, C5, C3, TP9, TP7, CP5, CP3, CP1, P7, P5, P3, PO7, PO3, O1, Fz, Pz, Oz, Fp2, AF4, AF8, F2, F4, F6, F8, FC2, FC4, FC6, FT8, FT10, C4, C6, C8, CP2, CP6, TP8, TP10, P2, P4, P6, P8, PO4, PO8 and O2, p = 0.0002, *d* = 0.0032, Fig 8M). Fast-gamma and harmalol concentration yielded two significant clusters, one at left fronto-temporal electrodes (F7, FT9 and FT7, p = 0.0032, *d* = 0.1479) and another at right frontal electrodes (AFz, AF4 and F2, p = 0.0050, *d* = 0.0785) (Fig 8N). Fast-gamma frequency band power increase and THH concentration formed one widespread large cluster spanning many different regions in both hemispheres (Fp1, AF7, F7, F3, FT9, FT7, FC5, FC3, T7, C5, C3, TP9, TP7, CP5, CP3, CP1, P5, P3, PO7, O1, Fz, Pz, Oz, Fp2, AF4, F2, F4, F6, F8, FC2, FC4, FC6, C4, C6, T8, CP2, TP8, TP10, P2, P4, P6, P8, PO4, PO8 and O2, p = 0.0002, *d* = 0.0501, Fig 8O). THH-OH however formed two marginally significant clusters with delta band power increase. These were at one left frontal electrode (F7, p = 0.0306, *d* = 0.2644,) and at one right central electrode (C6, p = 0.0408, *d* = 0.0269) (Fig 8P). Also, THH-OH and theta band power increase resulted significant at one right central electrode (C6, p = 0.0452, *d* = 0.2630, Fig 8Q).

### Psychometric Correlations

No significant correlations were found between HRS—Brazilian Version and spectral alterations in the frequency bands analyzed nor with Cmax, Tmax or AUC of any compound.

## Discussion

We found ayahuasca to induce a biphasic effect with changes in neural oscillations composed of decreases in alpha (8–13 Hz) power 50 minutes after ingestion and increases in the power of fast oscillatory activity after 75 to a 125 minutes, especially in the slow- and fast-gamma frequency bands (30–50 and 50–100 Hz, respectively). Spectral changes followed the time course of the main compounds in ayahuasca. DMT and harmine peaked before THH and harmaline. In the group average harmine peaked 50 (Fig 1D), while DMT after 75 minutes (Fig 1A) from ayahuasca ingestion. Harmaline reached a plateau after 100 (Fig 1E) while THH reached a plateau after 150 minutes (Fig 1C). This suggests DMT and harmine can be closely related to the early phase of the experience, measured as reduced power in the alpha band after 50 minutes (Fig 3B), while harmaline and THH can be more strongly associated with the later phase of gamma-band increases after 75 minutes from ingestion (Figs 5 and 6). This pharmacokinetic is in agreement with previous studies [7,38–40] and the EEG effects are in partial agreement with different studies that found alpha decreases [26,27] and gamma increases after ayahuasca ingestion [24,28]. This is the first report to evidence the biphasic profile of these oscillatory changes. Although no significant correlations were found with the subjective ratings obtained with the HRS, it is likely that these alterations in EEG power spectra are related to the subjective effects induced by ayahuasca and measured using this scale. One possible reason for the lack of correlations between EEG and HRS results is that this questionnaire has not been fully validated in a large sample yet.

### Alpha power decreases

Alpha band power reductions 50 minutes after ingestion were located at left parieto-occipital electrodes. This is consistent with increased visual cortex BOLD signal during ayahuasca visions [41]. The asymmetry observed here is also similar to some previous reports suggesting stronger effects in the left hemisphere [24]. Alpha asymmetries are however difficult to interpret given many methodological considerations [42]. Nevertheless, recent investigations still suggest alpha asymmetries in parietal cortex as important during attentional control [43] and memory retrieval [44].

Alpha reductions in posterior regions of the brain were also observed after i.v. administration of psilocybin, another 5HT-2A receptor agonist. Using dynamic causal modeling, it was suggested that activation of these receptors in the posterior cingulate cortex account for alpha decreases after psilocybin [45]. The crucial involvement of 5HT-2A receptors in the subjective experience induced by psilocybin has also been shown by attenuation of effects using the 5HT-2A antagonist ketanserin [46]. This same antagonist also blocked psilocybin induced effects on alpha oscillations, visual-evoked potentials and visual hallucinations [47]. Therefore, it is also likely that the alpha power decreases observed in the present study are due to DMT activation of cortical 5HT-2A receptors, whose density is high in parieto-occipital cortex [48].

### Gamma power increases

Increases in slow- (30–50 Hz) and fast-gamma (50–100 Hz) after 75 minutes from ayahuasca ingestion were significant at left fronto-temporal, left centro-parieto-occipital and right frontal cortices (Fig 5), while fast-gamma increases were significant in these same regions as well as right occipito-temporal electrodes (Fig 6). Slow-gamma power increases after ayahuasca have been reported previously in left occipito-temporo-parietal sites [24] and widespread increase in coherence was reported [28]. However, increases in the gamma range must be interpreted with caution due to possible interference from muscle artifacts [49]. In the present study, a series of pre-processing steps was adopted to minimize these. There are, thus, four reasons why the current results in the gamma range do not seem to be due to muscle artifacts. First, the amount of data-loss due to movement did not increase over time after ayahuasca administration, as would be expected if muscle activity was the source of the increases observed in the gamma band after 75 to 125 minutes from ingestion. Second, muscle activity spans a very broad frequency band (from 20–300 Hz [49]), which is inconsistent with the absence of effects in the beta band (13–30 Hz). Third, the topographic distribution of the effects in slow- and fast-gamma, with the former more concentrated in the left-hemisphere, are also hard to explain based on possible interference due to muscle artifacts. Fourth, we employed a very careful pre-processing with visual inspection of the tracings before and after ICA removal of components with a spectrum typical of muscle activity (very low power from 1–15 and a step increase from 15 Hz up). Furthermore, visual observation of the tracings during the live recordings made it possible to observe many instances of tracings with high-frequency activity but low amplitude while subjects were very still. These tracings are markedly different from the very large amplitude typical of movement artifacts when subjects move. Thus, it seems that the observed power increases in the gamma range after ayahuasca ingestion can be considered neural in origin. These can be important given the long interest in gamma frequency as a neural mechanism for binding of distributed neural representations in a cohesive subjective percept [50,51]. This includes synchronization in the parietal and frontal cortices critical for contour integration in the visual domain [52], processes of attention and memory [53] and synchronization in fronto-parietal networks which are important for the conscious activity culminating in a reportable subjective experience [54]1. These gamma increases are also found during other introspective states such as meditation [55] or lucid dreaming [56]. Remarkably, a recent report revealed that self-awareness can be induced during dreaming through frontal stimulation in the slow-gamma band [57]. Thus it is possible that these gamma power increases may be related to increased awareness of one's own internal psychological state, possibly including increased awareness of memories and intentions through potentiated visual imagery [41].

### Relevant alpha-gamma interactions

The alpha power decrease at occipito-parietal regions and the gamma power increase at frontal electrodes also closely match results obtained during emotional regulation by cognitive reappraisal [58]. This can have great implications to the understanding of ayahuasca's effect as related to increased awareness of one's emotions and how to deal with them, which was one reason mentioned by our volunteers to use ayahuasca. The observed pattern of combined changes in alpha and gamma at frontal and parieto-occipital electrodes closely resembles a report about insightful problem solving [59] and also the proposal of reduced alpha as diminishing inhibition and increased gamma as a sign of active processing in specific regions [60]. Given that in the ritual use of ayahuasca more than one dose is generally ingested with intervals around one hour or more, it is reasonable to expect that in such circumstances the second dose may induce an alpha decrease that may overlap in time with the gamma effects from the first dose. This may change the effects of ayahuasca in the brain not only in degree but in kind. Although repeated doses were investigated previously, this scenario was not reported, likely due to averaging power over 19 electrodes and low-pass filtering the data at 30 Hz [29].

### Plasma levels

The detected IAA (Fig 1B) can be attributed to ayahuasca ingestion because the method does not have significant matrix effects [31]. This corroborates previous evidence of partial MAO-A inhibition after ayahuasca intake [61], an important indicator of physical safety of ayahuasca consumption. Thus, after intake the metabolism of the tryptamines is shifted in favor of the less-efficient CYP enzymes. The consequence is detectable levels of DMT, DMT-NO, NMT and IAA in peripheral circulation (Fig 1A and 1B) due to simultaneous action of MAO-A and CYP enzymes. The positive correlation of IAA Cmax and AUC with age might suggest decreased inhibition of MAO activity in older subjects. However, in this case it would be expected to find decreasing DMT concentrations in older subjects, which was not the case. In fact, DMT Cmax and AUC correlations with age were also positive, although not statistically significant. The possible impact of the increased levels of IAA in the blood of older subjects after ayahuasca intake deserves more detailed study.

When using plasma concentrations as predictors of EEG spectral changes we were able to increase the signal to noise ratio and therefore better deal with the large variability observed between and within subjects. The emerging topographical distribution of significant effects thus assumes a more widespread distribution in the scalp. Alpha band decreases extended from left parieto-occipital only (Fig 3A) to centro-parieto and sometimes even frontal electrodes (Fig 7B, 7C and Fig 8A–8E). Slow- and fast-gamma results appear even more widespread through the whole scalp (Fig 7G–7N and Fig 8F–8O). Similar patterns were observed for the tryptamines (Fig 7) and the beta-carbolines (Fig 8), but with some important particularities which we discuss below. Some less intense and more localized effects also appeared in the delta, theta and beta bands regarding concentration of some specific compounds, discussed below.

### Tryptamines and the EEG

The most notable DMT effect seems to be reductions in alpha power. Using DMT concentration as a predictor of power changes extended the alpha cluster from left posterior parieto-occipital electrodes (Fig 3A) to a more broad parieto-central region (Fig 7B). Significant clusters were also found between DMT and gamma increases (Fig 7G and 7K), suggesting DMT also may contribute to effects beyond alpha decreases at 50 minutes. The small discrepancy between DMT peak at 75 and alpha decreases at 50 minutes may be explained by DMT's rapid and efficient transport into the brain [18,62] and active transport to intracellular vesicles [18,63].

The interaction between IAA, a naturally occurring compound in the metabolism of serotonin, and the alpha and gamma frequency bands reveal that the there is some degree of nonspecificity in the current analysis (Fig 7D, 7J and 7N). However, these effects were considerably less intense than those found for DMT, and the topographic distribution of these clusters is distinct than those found for the other tryptamines. Thus, it is likely that these were obtained indirectly by the correlation between IAA and DMT in the blood. Given the distinct topographical distributions, it also shows that the analysis was able to discern psychoactive from non-psychoactive compounds to a reasonable extent.

This brings the question of possible psychoactive roles for DMT-NO and NMT. The significant alpha power decreases estimated with the concentration of DMT-NO as a predictor variable reached left- and right-frontal electrodes, differentiating DMT-NO from DMT (Fig 7B and 7C). Given that IAA also formed a small cluster at left frontal electrodes (Fig 7D) part of the alpha cluster for DMT-NO may not be particularly important. However, DMT-NO also formed larger clusters than DMT in the gamma bands. The slow- and fast-gamma clusters detected with DMT-NO reached temporoparietal electrodes (Fig 7H and 7L), further differentiating it from DMT (Fig 7G and 7K). It is hard to explain more widespread clusters with the metabolite than with the parent compound if not by the metabolite having some effect at the central nervous system. But in the absence of reports regarding possible psychoactive effects of DMT-NO, the current data is inconclusive regarding psychoactivity of this compound. NMT, on the other hand, did not form any significant cluster with the alpha band, but did so with the slow- and fast-gamma bands (Fig 7I and 7M). These were more concentrated in bilateral frontal and parieto-occipital areas, differentiating NMT from DMT and DMT-NO (Fig 7G, 7H, 7K and 7L). Evidence about NMT psychoactivity is scarce, but specialized internet forums report NMT to be psychoactive if smoked or combined with MAO inhibitors with a particular quality that makes NMT distinguishable from DMT [64].

### Beta-carbolines and the EEG

The strong and widespread clusters found between beta-carbolines circulating levels and spectral changes in the EEG (Fig 8) suggests these compounds do contribute to the effects of ayahuasca in the brain, besides being peripheral MAO-A inhibitors. The possible involvement of the beta-carbolines in the central effects of ayahuasca is further corroborated by their presence in many different psychoactive plants, including *Peganum harmala* seeds, from where they derive their names. Many of these plants are used by oral ingestion and other routes [2,3] and it is known that some forms of ayahuasca can be prepared exclusively with *B*. *caapi* [3,65] [66], corroborated by a report of ayahuasca samples not containing DMT [9].

### Harmine and the EEG

When using the concentration of harmine (and also of harmol) as a predictor of EEG power spectral changes, the alpha cluster was virtually identical to that of DMT (Figs 7B and 8A, respectively). However, in the slow-gamma band harmine formed a significant cluster in the right-parietal cortex (Fig 8F) whereas DMT did not (Fig 7G), while in the fast-gamma harmine predicted significant power changes in almost all the cortex surface. This is hard to explain as indirect effects solely based on correlations between DMT and harmine concentrations in plasma, with some effects being probably linked to harmine's pharmacological actions in the brain, mostly through central MAO-A inhibition. Indeed harmine is such an effective central MAO-A inhibitor that it became the molecule of choice in positron emission tomography (PET) studies about MAO-A in the brain [67,68]. Furthermore, harmine also has moderate affinity for 5HT-2A receptors as well as actions on 5HT-2C, I2 and the dopamine transporter [69], expanding the mechanisms of action of ayahuasca in the CNS.

Harmine has been shown previously to be psychoactive in i.v. doses of 200 mg or more, even though qualitatively different from LSD or mescaline [70]. In another study, 10 to 20 mg/kg i.m. harmine was reported as having effects qualitatively similar to LSD or mescaline [71]. At doses of 120 mg orally, it has been reported to induce "barely-perceptible sedative effects" [19]. Considering the average amount of harmine orally ingested in the present study was 300 mg (with the maximum estimated at almost 400), a contribution to the overall psychoactive effects of ayahuasca is likely. Furthermore, recent experimental evidence from human liver microsomes suggests the existence of additional metabolic pathways for harmine [72] which have not been studied in the context of ayahuasca ingestion.

### THH and the EEG

THH plasma levels were associated with changes in alpha, slow- and fast-gamma bands (Figs 8E, 7J and 7O, respectively) forming a pattern similar to that of harmine and DMT. But the alpha cluster resulting from THH concentration as a predictor was even more widespread, reaching a few electrodes in the right hemisphere (Fig 8E). THH was also associated with intense and widespread gamma activity, especially in the fast-gamma band (Fig 8O). The obtained pattern for THH-OH, on the other hand, was very different. No clusters were found for alpha and gamma bands, with some marginally significant effects localized to individual electrodes in the delta and theta bands (Fig 8P and 8Q, respectively). Given that these effects in delta and theta are spatially restricted and orders of magnitudes smaller than those for the other beta-carbolines and frequency bands, it seems unlikely that they play any major role in the acute effects of ayahuasca.

The only available report about the psychoactive properties of pure THH we are aware of was done with only one subject. After oral ingestion of 300 mg of racemic THH the subjective effects were rated as similar to 100 mg harmaline [10]. Possible effects of THH in the brain include moderate affinity for 5HT-2A receptors [73], inhibition of serotonin reuptake and MAO-A inhibition [74]. In the current study we found very low levels of THH-OH in the blood. Although THH half-life has previously been shown to be almost 9 hours [7], the possible formation of other metabolites through different enzymatic pathways is supported by data obtained after ingestion of ayahuasca where THH recovery in 24 h urine as THH and THH-OH was as low as 9% [61]. A possible new metabolic pathway was recently demonstrated through oxidation into bioactive compounds such as harman and norharman by heme peroxidases [75]. Possible effects of these compounds in the brain remain unknown.

### Harmaline and the EEG

Levels of harmaline in the blood were significantly associated with strong effects in the alpha, slow- and fast-gamma bands (Fig 8C, 8H and 8M). These were stronger as compared to harmine or DMT, and especially intense in the fast-gamma range (Fig 8M). The alpha cluster found with harmaline concentration was larger and more intense than the one found for DMT, suggesting harmaline contributes to the effects of ayahuasca in the brain. Although harmaline is usually found in small amounts in ayahuasca preparations [9], it is a likely candidate for significantly contributing to the effects of ayahuasca. It can induce psychoactive and hallucinogenic effects after oral or i.v. administration [10] with the dose necessary for psychoactivity being determined as 4 mg/kg. It has also been reported as being orally psychoactive between 300–400 mg [76]. Although these are higher than the estimated average dose in the present study, ingestion of harmaline with harmine and THH has not been systematically tested. The psychoactive effects of harmaline can be due to affinity with different types of 5HT receptors, including 5HT-2A and the serotonin transporter, among other targets [77,78]. Drug discrimination studies in rats showed harmaline to substitute the psychedelic DOM [79], which fully cross-substitute with DMT [80]. Furthermore, harmaline potentiates 5-MeO-DMT and 5-OH-DMT [81,82]. This was corroborated by the only report we could find of ingestion of harmaline with DMT being described briefly as eliciting effects similar to ayahuasca (Bigwood apud [19]).

Furthermore, harmaline's Tmax significantly correlated with the time for emesis (Fig 1F) while DMT, THH and harmine did not. This is in agreement with nausea being a common effect after ingestion of *Peganum harmala*, which contains high concentrations of harmaline [83]. Also, in previous reports of harmaline intake, strong nausea and vomiting were mentioned as common ([10], Bigwood apud [19]), while lack of such an effect after oral ingestion of DMT and harmine was noted [19]. Thus harmaline seems to be crucially involved in the emetic properties of ayahuasca.

### Emesis

Given that nausea and vomiting can be undesired and challenging in palliative care [84], their occurrence has been considered an undesirable side-effect of ayahuasca [85]. When using ayahuasca lyophilized, vomiting has been reported to be uncommon [26,85] and when it occurred it lead researchers to discard data from the respective subjects (reaching a considerable 30%) [29]. Although it was suggested that vomiting may interfere with bioavailability [85], we did not find any significant correlations between the time to vomit with Cmax or AUC of any of the 10 compounds analyzed in blood. Given harmaline's positive correlation with the time to vomit, it is possible that the absorption of DMT and harmine, which peak faster in the blood, are not critically affected by emesis in ayahuasca. THH, on the other hand, may be affected given it's slower time course which extends beyond the monitoring done in the present investigation.

In contrast with biomedical concepts, nausea and vomiting assume central status among indigenous cultures who developed and use ayahuasca and also do so in ayahuasca religions. A striking example are the Matsigenka, whose name for ayahuasca is *kamarampi*, literally "vomiting medicine" [86]. Participants in ayahuasca healing sessions report vomiting as an important part of the cleansing process and claim it helps them to emotionally heal [87]. Phenomenological studies proposed nausea and purging as crucial to direct attention to bodily states, to help confront challenges posed by bodily malaise and to learn how to overcome it [88].

It is therefore crucial to consider that nausea and vomiting can be psychogenic in origin and thus can be involved in the emotional experience induced by ayahuasca ingestion. Awareness of visceral stimuli has been proposed as part of the formation of emotional and affective states [89,90] and this can occur through bottom-up information from the viscera to a brain circuitry involving the amygdala, frontal cortex and insula [89–92]. The anterior insula has specifically been proposed as a site of integration of neural information related to the awareness of interoceptive and affective states [90] and emotional awareness [93]. The only single-photon emission computerized tomography (SPECT) study to date with ayahuasca showed increased activation in the amygdala, regions of the frontal cortex and anterior insula [94]. Although no information regarding nausea and/or vomiting was provided, it is suggestive of increased bodily awareness during ayahuasca, possibly leading to conscious perception of one own's emotional and affective state. Remarkably, most of the EEG spectral changes in alpha and gamma bands were located near the temporo-parietal junction, an area involved in multisensory integration originating the sensation of self and bodily location [95,96]. This may be one of the most fundamental features of ordinary consciousness, i.e., the sense of being a cohesive self in precise locations in space. It depends on visual and tactile information [95] most likely altered by ayahuasca, thus changing the sensation of self. As harmaline was found to be strongly related with increases in fast-gamma power in widespread cortical regions, it possibly links nausea and emesis with emotion, affection and the sensation of self. In agreement with this, some of the volunteers claimed to use ayahuasca to get insights regarding their emotions and interpersonal relationships.

### Placebo effects

One possible limitation of the present study is the absence of blinding to control for placebo effects. Although the lyophilizate was developed and employed in order to account for placebo effects in pharmacological research with ayahuasca [85], the strong subjective effects, visions and physical sensations makes it almost certain that blinding will be broken as soon as the effects become noticeable, a problem aggravated when studying experienced volunteers. Therefore, the double-blind design has dubious practical value in studying a psychoactive whose effects the subjects become aware of. The onset of EEG effects presently measured were faster than those reported with lyophilized ayahuasca [26,29], suggesting slower absorption dynamics when using the lyophilizate. Furthermore, when studying the brain's oscillatory activity, placebo sessions as controls introduce biases of arousal, vigilance state (most especially drowsiness during the placebo condition), motivation and attention. Drowsiness can be an important confounding factor, especially when results point to decreases in delta activity [26,27]. The likelihood of the present EEG results being due to placebo effects is small because of parallels with the plasma results.

The present design thus seems to make feasible a more ecologically valid approach to the neuroscientific study of ayahuasca. As was recently argued, it is important for biomedical and pharmacological studies to thoroughly consider the social, cultural and complex chemical composition of ayahuasca to avoid altering the object of their inquiry as a function of methodological imperatives [97]. In this regard, the current study is also limited to the extent that it ignores many important elements of ritual ayahuasca use, including music, singing, communal experiences and freedom to act when not required to stay still in benefit of the EEG.

## Conclusion

The present results reveal acute biphasic effects of ayahuasca in the brain. Changes were mostly constituted of alpha decreases in the left centro-parieto-occipital cortex after 50 minutes and gamma band increases in frontal, parietal and temporal areas 75 to a 125 minutes after ingestion. These effects are associated with concentrations of tryptamines and beta-carbolines in the blood, suggesting a modified state of consciousness induced by interactions among many ayahuasca compounds. This is in agreement with the understanding of Schultes and Hofmann that "The hallucinogenic effects of ayahuasca preparations may be the result of the combined activity of harmine and its derivatives with dimethyltryptamine and other tryptamine derivatives which were found in certain admixtures to ayahuasca such as *Banisteriopsis rusbyana* and *Psychotria viridis*. The MAO inhibitors harmine and harmaline may enhance the effects of tryptamines. It should, however, be borne in mind that the narcotic is still hallucinogenic even when, as often is the case, it is prepared exclusively from species of Banisteriopsis without the tryptamine additives." [3]. This comprehensive approach to ayahuasca pharmacology and it's cognitive, affective and emotional effects is relevant to the ritual use of ayahuasca and to studies about its therapeutic potentials, including treatment of depression, anxiety, post-traumatic stress disorder and drug dependence.

## References

[1] Metzner R. Hallucinogenic drugs and plants in psychotherapy and shamanism. J of Psychoactive Drugs. 1998;30(4):333–41. 9924839 10.1080/02791072.1998.10399709

[2] Schultes RE. Hallucinogens of plant origin. Science. 1969 1 17;163(3864):245–54. 4883616 10.1126/science.163.3864.245

[3] Schultes RE, Hofmann A. The botany and chemistry of hallucinogens. …: Charles C Thomas 267pp Hallucinogens …. 1973.

[4] Labate BC, Feeney K. Ayahuasca and the process of regulation in Brazil and internationally: Implications and challenges. International Journal of Drug Policy. 2012 8 17;23:154–61. 10.1016/j.drugpo.2011.06.006 21856141

[5] Rivier L, Lindgren J-E. “Ayahuasca,” the South American hallucinogenic drink: An ethnobotanical and chemical investigation. Econ Bot. 1972 4;26(2):101–29.

[6] Mcilhenny EH, Pipkin KE, Standish LJ, Wechkin HA, Strassman RJ, Barker SA. Direct analysis of psychoactive tryptamine and harmala alkaloids in the Amazonian botanical medicine ayahuasca by liquid chromatography-electrospray ionization-tandem mass spectrometry. Journal of Chromatography A. 2009 12 18;1216(51):8960–8. 10.1016/j.chroma.2009.10.088 19926090

[7] Callaway J, McKenna DJ, Grob CS, Brito GS, Raymon LP, Poland RE, et al Pharmacokinetics of Hoasca alkaloids in healthy humans. Journal of Ethnopharmacology. 1999 6;65(3):243–56. 10404423 10.1016/s0378-8741(98)00168-8

[8] Callaway J, Brito GS, Neves ES. Phytochemical analyses of Banisteriopsis caapi and Psychotria viridis. J of Psychoactive Drugs. 2005 6;37(2):145–50. 16149327 10.1080/02791072.2005.10399795

[9] Callaway J. Various alkaloid profiles in decoctions of Banisteriopsis caapi. J of Psychoactive Drugs. 2005 6;37(2):151–5. 16149328 10.1080/02791072.2005.10399796

[10] Naranjo C. Psychotropic properties of the harmala alkaloids. Ethnopharmacologic Search for Psychoactive Drugs US Public Health Service Publication; 1967. 1 p.

[11] Naranjo C. Ayahuasca, caapi, yage. Psychotropic properties of the harmala alkaloids. Psychopharmacol Bull. 1967 12;4(3):16–7. 5615550

[12] Naranjo C. Ayahuasca Imagery and the therapeutic property of the harmala Alkaloids. Journal of mental imagery. 1987;11(2):131–6.

[13] Szára S. DMT at fifty. Neuropsychopharmacol Hung. 2007 12 1;9(4):201–5. 18510265

[14] Holmstedt B, Lindgren J. Chemical constituents and pharmacology of South American snuffs. Psychopharmacol Bull. 1967 12;4(3):16. 5615549

[15] McKenna DJ, Towers GH, Abbott FS. Monoamine oxidase inhibitors in South American hallucinogenic plants Part 2: Constituents of orally-active Myristicaceous hallucinogens. Journal of Ethnopharmacology. 1984 11;12(2):179–211. 6521493 10.1016/0378-8741(84)90048-5

[16] McKenna DJ, Towers GH, Abbott FS. Monoamine oxidase inhibitors in South American hallucinogenic plants: tryptamine and beta-carboline constituents of ayahuasca. Journal of Ethnopharmacology. 1984 4 1;10(2):195–223. 6587171 10.1016/0378-8741(84)90003-5

[17] Samoylenko V, Rahman MM, Tekwani BL, Tripathi LM, Wang Y-H, Khan SI, et al Banisteriopsis caapi, a unique combination of MAO inhibitory and antioxidative constituents for the activities relevant to neurodegenerative disorders and Parkinson's disease. Journal of Ethnopharmacology. 2010 2 3;127(2):357–67. 10.1016/j.jep.2009.10.030 19879939 PMC2828149

[18] Frecska E, Szabo A, Winkelman MJ, Luna LE, McKenna DJ. A possibly sigma-1 receptor mediated role of dimethyltryptamine in tissue protection, regeneration, and immunity. J Neural Transm. 2013 9;120(9):1295–303. 10.1007/s00702-013-1024-y 23619992

[19] Ott J. Pharmahuasca: human pharmacology of oral DMT plus harmine. J of Psychoactive Drugs. 1999 4;31(2):171–7. 10438001 10.1080/02791072.1999.10471741

[20] Ott J. Pharmahuasca, Anahuasca and Vinho da Jurema: Human Pharmacology of Oral DMT Plus Harmine. yearbook for Ethnomedicine. 1997;:1–13.

[21] Smith RL, Canton H, Barrett RJ, Sanders-Bush E. Agonist properties of N,N-dimethyltryptamine at serotonin 5-HT2A and 5-HT2C receptors. Pharmacol Biochem Behav. 1998 11 1;61(3):323–30. 9768567 10.1016/s0091-3057(98)00110-5

[22] Fontanilla D, Johannessen M, Hajipour AR, Cozzi NV, Jackson MB, Ruoho AE. The hallucinogen N,N-dimethyltryptamine (DMT) is an endogenous sigma-1 receptor regulator. Science. 2009 2 13;323(5916):934–7. 10.1126/science.1166127 19213917 PMC2947205

[23] Wallach JV. Endogenous hallucinogens as ligands of the trace amine receptors: a possible role in sensory perception. Medical Hypotheses. 2009;72(1):91–4. 10.1016/j.mehy.2008.07.052 18805646

[24] Don N, McDonough B, Moura G, Warren C, Kawanishi K, Tomita H, et al Effects of Ayahuasca on the human EEG. Phytomedicine. 1998;5(2):87–96. 10.1016/S0944-7113(98)80003-2 23195759

[25] Hoffmann E, Hesselink JMK, Barbosa Y-WDS. Effects of a Psychedelic, Tropical Tea, Ayahuasca, on the Electroencephalographic (EEG) Activity of the Human Brain During a Shamanistic Ritual. MAPS Bulletin. 2001 6 19;11(1):25–30.

[26] Riba J, Anderer P, Morte A, Urbano G, Jané F, Saletu B, et al Topographic pharmaco-EEG mapping of the effects of the South American psychoactive beverage ayahuasca in healthy volunteers. Br J Clin Pharmacol. 2002 6 1;53(6):613–28. 12047486 10.1046/j.1365-2125.2002.01609.x PMC1874340

[27] Riba J, Anderer P, Jané F, Saletu B, Barbanoj MJ. Effects of the South American psychoactive beverage ayahuasca on regional brain electrical activity in humans: a functional neuroimaging study using low-resolution electromagnetic tomography. Neuropsychobiology. 2004;50(1):89–101. 15179026 10.1159/000077946

[28] Stuckey DE, Lawson R, Luna LE. EEG gamma coherence and other correlates of subjective reports during ayahuasca experiences. J of Psychoactive Drugs. 2005 6 1;37(2):163–78. 16149330 10.1080/02791072.2005.10399798

[29] Santos Dos RG, Grasa E, Valle M, Ballester MR, Bouso JC, Nomdedéu JF, et al Pharmacology of ayahuasca administered in two repeated doses. Psychopharmacology. 2012 2;219(4):1039–53. 10.1007/s00213-011-2434-x 21842159

[30] Johnson M, Richards W, Griffiths RR. Human hallucinogen research: guidelines for safety. Journal of Psychopharmacology. 2008 8 1;22(6):603–20. 10.1177/0269881108093587 18593734 PMC3056407

[31] Mcilhenny EH, Riba J, Barbanoj MJ, Strassman RJ, Barker SA. Methodology for determining major constituents of ayahuasca and their metabolites in blood. Biomedical chromatography: BMC. 2012 3 1;26(3):301–13. 10.1002/bmc.1657 21710581

[32] Mizumoto S, Silveira DX, Barbosa PCR. Hallucinogen Rating Scale (HRS)-A Brazilian version: translation and cross-cultural adaptation. Revista de Psiquiatria Clinica. 2011 12 27;38(6):231–7.

[33] Delorme A, Makeig S. EEGLAB: an open source toolbox for analysis of single-trial EEG dynamics including independent component analysis. Journal of Neuroscience Methods. 2004 3 15;134(1):9–21. 15102499 10.1016/j.jneumeth.2003.10.009

[34] Perrin F, Pernier J, Bertrand O, Echallier JF. Spherical splines for scalp potential and current density mapping. Electroencephalogr Clin Neurophysiol. 1989 2;72(2):184–7. 2464490 10.1016/0013-4694(89)90180-6

[35] Oostenveld R, Fries P, Maris E, Schoffelen J-M. FieldTrip: Open Source Software for Advanced Analysis of MEG, EEG, and Invasive Electrophysiological Data. Computational Intelligence and Neuroscience. 2011;2011:1–9. 10.1155/2011/156869 21253357 PMC3021840

[36] Mitra PP, Pesaran B. Analysis of dynamic brain imaging data. Biophys J. 1999 2 1;76(2):691–708. 9929474 10.1016/S0006-3495(99)77236-X PMC1300074

[37] Maris E, Oostenveld R. Nonparametric statistical testing of EEG- and MEG-data. Journal of Neuroscience Methods. 2007 8 15;164(1):177–90. 17517438 10.1016/j.jneumeth.2007.03.024

[38] Callaway J. Fast and slow metabolizers of Hoasca. J of Psychoactive Drugs. 2005 6;37(2):157–61. 16149329 10.1080/02791072.2005.10399797

[39] Callaway J, Raymon LP, Hearn WL, McKenna DJ, Grob CS, Brito GS, et al Quantitation of N,N-dimethyltryptamine and harmala alkaloids in human plasma after oral dosing with ayahuasca. J Anal Toxicol. 1996 10;20(6):492–7. 8889686 10.1093/jat/20.6.492

[40] Grob CS, McKenna DJ, Callaway J, Brito GS, Neves ES, Oberlaender G, et al Human psychopharmacology of hoasca, a plant hallucinogen used in ritual context in Brazil. The Journal of Nervous and mental disease. 1996 2;184(2):86–94. 8596116 10.1097/00005053-199602000-00004

[41] De Araujo DB, Ribeiro S, Cecchi GA, Carvalho FM, Sanchez TA, Pinto JP, et al Seeing with the eyes shut: neural basis of enhanced imagery following Ayahuasca ingestion. Hum Brain Mapp. 2012 11;33(11):2550–60. 10.1002/hbm.21381 21922603 PMC6870240

[42] Davidson RJ. EEG measures of cerebral asymmetry: conceptual and methodological issues. Int J Neurosci. 1988 3;39(1–2):71–89. 3290140 10.3109/00207458808985694

[43] Alfonso M-R, Miquel T-F, Xavier B, Blanca A-S. Resting parietal electroencephalogram asymmetries and self-reported attentional control. Clin EEG Neurosci. SAGE Publications; 2013 7;44(3):188–92. 10.1177/1550059412465871 23545247

[44] Nelson SM, McDermott KB, Wig GS, Schlaggar BL, Petersen SE. The critical roles of localization and physiology for understanding parietal contributions to memory retrieval. The Neuroscientist: a review journal bringing neurobiology, neurology and psychiatry. 2013 12;19(6):578–91. 10.1177/1073858413492389 23778789

[45] Muthukumaraswamy SD, Carhart-Harris R, Moran RJ, Brookes MJ, Williams TM, Errtizoe D, et al Broadband Cortical Desynchronization Underlies the Human Psychedelic State. Journal of Neuroscience. 2013 9 18;33(38):15171–83. 10.1523/JNEUROSCI.2063-13.2013 24048847 PMC6618409

[46] Vollenweider FX, Vollenweider-Scherpenhuyzen M, Babler A, Vogel H, Hell D. Psilocybin induces schizophrenia-like psychosis in humans via a serotonin-2 agonist action. Neuroreport. 1998;9(17):3897–902. 9875725 10.1097/00001756-199812010-00024

[47] Kometer M, Schmidt A, Jäncke L, Vollenweider FX. Activation of Serotonin 2A Receptors Underlies the Psilocybin-Induced Effects on α Oscillations, N170 Visual-Evoked Potentials, and Visual Hallucinations. Journal of Neuroscience. 2013 6 19;33(25):10544–51. 10.1523/JNEUROSCI.3007-12.2013 23785166 PMC6618596

[48] Erritzoe D, Frokjaer VG, Haugbol S, Marner L, Svarer C, Holst K, et al Brain serotonin 2A receptor binding: relations to body mass index, tobacco and alcohol use. NeuroImage. 2009 5 15;46(1):23–30. 10.1016/j.neuroimage.2009.01.050 19457377

[49] Muthukumaraswamy SD. High-frequency brain activity and muscle artifacts in MEG/EEG: a review and recommendations. Front Hum Neurosci. Frontiers; 2013;7. 10.3389/fnhum.2013.00138 PMC3625857 23596409

[50] Fries P, Nikolic D, Singer W. The gamma cycle. Trends in Neurosciences. 2007 7 1;30(7):309–16. 17555828 10.1016/j.tins.2007.05.005

[51] Rodriguez E, George N, Lachaux JP, Martinerie J, Renault B, Varela FJ. Perception's shadow: long-distance synchronization of human brain activity. Nature. 1999 2 4;397(6718):430–3. 9989408 10.1038/17120

[52] Castellano M, Plöchl M, Vicente R, Pipa G. Neuronal oscillations form parietal/frontal networks during contour integration. Front Integr Neurosci. 2014;8:64 10.3389/fnint.2014.00064 25165437 PMC4131516

[53] Jensen O, Kaiser J, Lachaux J-P. Human gamma-frequency oscillations associated with attention and memory. Trends in Neurosciences. 2007 7;30(7):317–24. 17499860 10.1016/j.tins.2007.05.001

[54] Dehaene S, Changeux J-P. Experimental and theoretical approaches to conscious processing. Neuron. 2011 4 28;70(2):200–27. 10.1016/j.neuron.2011.03.018 21521609

[55] Lutz A, Greischar LL, Rawlings NB, Ricard M, Davidson RJ. Long-term meditators self-induce high-amplitude gamma synchrony during mental practice. Proc Natl Acad Sci USA. 2004 11 16;101(46):16369–73. 15534199 10.1073/pnas.0407401101 PMC526201

[56] Voss U, Holzmann R, Tuin I, Hobson JA. Lucid dreaming: a state of consciousness with features of both waking and non-lucid dreaming. Sleep. 2009 9 1;32(9):1191–200. 19750924 10.1093/sleep/32.9.1191 PMC2737577

[57] Voss U, Holzmann R, Hobson A, Paulus W, Koppehele-Gossel J, Klimke A, et al Induction of self awareness in dreams through frontal low current stimulation of gamma activity. Nat Neurosci. 2014 6;17(6):810–2. 10.1038/nn.3719 24816141

[58] Popov T, Steffen A, Weisz N, Miller GA, Rockstroh B. Cross-frequency dynamics of neuromagnetic oscillatory activity: two mechanisms of emotion regulation. Psychophysiology. 2012 12;49(12):1545–57. 10.1111/j.1469-8986.2012.01484.x 23074972

[59] Sandkühler S, Bhattacharya J. Deconstructing insight: EEG correlates of insightful problem solving. PLoS ONE. 2008;3(1):e1459 10.1371/journal.pone.0001459 18213368 PMC2180197

[60] Jensen O, Mazaheri A. Shaping functional architecture by oscillatory alpha activity: gating by inhibition. Front Hum Neurosci. 2010;4:186 10.3389/fnhum.2010.00186 21119777 PMC2990626

[61] Riba J, Mcilhenny EH, Valle M, Bouso JC, Barker SA. Metabolism and disposition of N,N-dimethyltryptamine and harmala alkaloids after oral administration of ayahuasca. Drug Test Anal. 2012 7;4(7–8):610–6. 10.1002/dta.1344 22514127

[62] Vitale AA, Pomilio AB, Cañellas CO, Vitale MG, Putz EM, Ciprian-Ollivier J. In vivo long-term kinetics of radiolabeled n,n-dimethyltryptamine and tryptamine. J Nucl Med. 2011 6 1;52(6):970–7. 10.2967/jnumed.110.083246 21622895

[63] Cozzi NV, Gopalakrishnan A, Anderson LL, Feih JT, Shulgin AT, Daley PF, et al Dimethyltryptamine and other hallucinogenic tryptamines exhibit substrate behavior at the serotonin uptake transporter and the vesicle monoamine transporter. J Neural Transm. 2009 12 1;116(12):1591–9. 10.1007/s00702-009-0308-8 19756361

[64] Nexus D. Entheogenic effects of DMT. Available: https://www.dmt-nexus.me/forum/default.aspx?g=posts&m=300323#post300323.

[65] Rodd R. Reassessing the cultural and psychopharmacological significance of Banisteriopsis caapi: preparation, classification and use among the Piaroa of Southern Venezuela. J of Psychoactive Drugs. Taylor & Francis; 2008;40(3):301–7. 19004422 10.1080/02791072.2008.10400645

[66] Davis W. One River. Random House; 2014. 1 p.

[67] Ginovart N, Meyer JH, Boovariwala A, Hussey D, Rabiner EA, Houle S, et al Positron emission tomography quantification of [11C]-harmine binding to monoamine oxidase-A in the human brain. J Cereb Blood Flow Metab. 2006 3;26(3):330–44. 16079787 10.1038/sj.jcbfm.9600197

[68] Sacher J, Rabiner EA, Clark M, Rusjan P, Soliman A, Boskovic R, et al Dynamic, adaptive changes in MAO-A binding after alterations in substrate availability: an in vivo [11C]-harmine positron emission tomography study. Journal of Cerebral Blood Flow &amp; Metabolism. Nature Publishing Group; 2011 12 21 ; 32(3):443–6. 22186668 10.1038/jcbfm.2011.184 PMC3293124

[69] Brierley DI, Davidson C. Developments in harmine pharmacology—implications for ayahuasca use and drug-dependence treatment. Prog Neuropsychopharmacol Biol Psychiatry. 2012 12 3;39(2):263–72. 10.1016/j.pnpbp.2012.06.001 22691716

[70] Pennes HH, Hoch PH. Psychotomimetics, clinical and theoretical considerations: harmine, Win-2299 and nalline. Am J Psychiatry. 1957 4;113(10):887–92. 13402982 10.1176/ajp.113.10.887

[71] Naranjo P. Estudio comparativo de la harmina, la dietilamida del ácido lisérgico (LSD-25) y la mescalina. Revista de la Confederación Médica Panamericana. 1959;6:1–8.

[72] Zhao T, Zheng S-S, Zhang B-F, Li Y-Y, Bligh SWA, Wang C-H, et al Metabolic pathways of the psychotropic-carboline alkaloids, harmaline and harmine, by liquid chromatography/mass spectrometry and NMR spectroscopy. Food Chem. 2012 9 15;134(2):1096–105. 10.1016/j.foodchem.2012.03.024 23107733

[73] Glennon RA, Dukat M, Grella B, Hong SS. Binding of β-carbolines and related agents at serotonin (5-HT_2_ and 5-HT_1A_), dopamine (D_2_) and benzodiazepine …. Drug and alcohol …. 2000;60(2):121–32. 10.1016/s0376-8716(99)00148-9 10940539

[74] Airaksinen MM, Kari I. beta-Carbolines, psychoactive compounds in the mammalian body. Part II: Effects. Med Biol. 1981 8;59(4):190–211. 6121956

[75] Herraiz T, Galisteo J. Naturally-occurring tetrahydro-β-carboline alkaloids derived from tryptophan are oxidized to bioactive β-carboline alkaloids by heme peroxidases. Biochem Biophys Res Commun. 2014 8 15;451(1):42–7. 10.1016/j.bbrc.2014.07.047 25035927

[76] Shulgin AT. Profile of Psychedelic Drugs. 4. Harmaline. Journal of Psychedelic drugs. 1977 10 27;9(1):79–80. 10.1080/02791072.1980.10471557 7392062

[77] Grella B, Teitler M, Smith C, Herrick-Davis K, Glennon RA. Binding of beta-carbolines at 5-HT(2) serotonin receptors. Bioorg Med Chem Lett. 2003 12 15;13(24):4421–5. 14643338 10.1016/j.bmcl.2003.09.027

[78] Glennon RA, Grella B, Tyacke RJ, Lau A, Westaway J, Hudson AL. Binding of beta-carbolines at imidazoline I2 receptors: a structure-affinity investigation. Bioorg Med Chem Lett. 2004 2 23;14(4):999–1002. 15013009 10.1016/j.bmcl.2003.11.078

[79] Grella B, Dukat M, Young R, Teitler M, Herrick-Davis K, Gauthier CB, et al Investigation of hallucinogenic and related beta-carbolines. Drug and Alcohol Dependence. 1998 4 1;50(2):99–107. 9649961 10.1016/s0376-8716(97)00163-4

[80] Gatch MB, Rutledge MA, Carbonaro T, Forster MJ. Comparison of the discriminative stimulus effects of dimethyltryptamine with different classes of psychoactive compounds in rats. Psychopharmacology. 2009 7 1;204(4):715–24. 10.1007/s00213-009-1501-z 19288085 PMC2865430

[81] Jiang X-L, Shen H-W, Mager DE, Yu A-M. Pharmacokinetic interactions between monoamine oxidase A inhibitor harmaline and 5-methoxy-N,N-dimethyltryptamine, and the impact of CYP2D6 status. Drug Metab Dispos. 2013 5;41(5):975–86. 10.1124/dmd.112.050724 23393220 PMC3629804

[82] Shen H-W, Jiang X-L, Winter JC, Yu A-M. Psychedelic 5-Methoxy-N,N-Dimethyltryptamine: Metabolism, Pharmacokinetics, Drug Interactions, and Pharmacological Actions. Curr Drug Metab. 2010 10;11(8):659–66. 20942780 10.2174/138920010794233495 PMC3028383

[83] Herraiz T, González D, Ancín-Azpilicueta C, Arán VJ, Guillén H. beta-Carboline alkaloids in Peganum harmala and inhibition of human monoamine oxidase (MAO). Food Chem Toxicol. 2010 3;48(3):839–45. 10.1016/j.fct.2009.12.019 20036304

[84] Keeley PW. Nausea and vomiting. Medicine. 2011.

[85] Riba J, Barbanoj MJ. Bringing ayahuasca to the clinical research laboratory. J of Psychoactive Drugs. 2005 6 1;37(2):219–30. 16149336 10.1080/02791072.2005.10399804

[86] Shepard GH. Psychoactive plants and ethnopsychiatric medicines of the Matsigenka. J of Psychoactive Drugs. 1998 10;30(4):321–32. 9924838 10.1080/02791072.1998.10399708

[87] Schmid JT. Healing with Ayahuasca: Notes on Therapeutic Rituals and Effects in European Patients Treating Their Diseases The Therapeutic Use of Ayahuasca. Berlin, Heidelberg: Springer Berlin Heidelberg; 2014 pp. 77–93.

[88] Shanon B. Moments of Insight, Healing, and Transformation: A Cognitive Phenomenological Analysis In: Labate BC, Cavnar C, editors. The Therapeutic Use of Ayahuasca. Springer; 2013 pp. 59–75.

[89] Mayer EA. Gut feelings: the emerging biology of gut-brain communication. Nat Rev Neurosci. 2011 8;12(8):453–66. 10.1038/nrn3071 21750565 PMC3845678

[90] Singer T, Critchley HD, Preuschoff K. A common role of insula in feelings, empathy and uncertainty. Trends in Cognitive Sciences. 2009 8;13(8):334–40. 10.1016/j.tics.2009.05.001 19643659

[91] Berntson GG, Sarter M, Cacioppo JT. Ascending visceral regulation of cortical affective information processing. Eur J Neurosci. 2003 10;18(8):2103–9. 14622171 10.1046/j.1460-9568.2003.02967.x

[92] Napadow V, Sheehan JD, Kim J, Lacount LT, Park K, Kaptchuk TJ, et al The brain circuitry underlying the temporal evolution of nausea in humans. Cerebral Cortex. Oxford University Press; 2013 4;23(4):806–13. 10.1093/cercor/bhs073 22473843 PMC3593575

[93] Gu X, Hof PR, Friston KJ, Fan J. Anterior insular cortex and emotional awareness. J Comp Neurol. 2013 10 15;521(15):3371–88. 10.1002/cne.23368 23749500 PMC3999437

[94] Riba J, Romero S, Grasa E, Mena E, Carrió I, Barbanoj MJ. Increased frontal and paralimbic activation following ayahuasca, the pan-Amazonian inebriant. Psychopharmacologia. 2006 5;186(1):93–8. 10.1007/s00213-006-0358-7 16575552

[95] Ionta S, Heydrich L, Lenggenhager B, Mouthon M, Fornari E, Chapuis D, et al Multisensory mechanisms in temporo-parietal cortex support self-location and first-person perspective. Neuron. 2011 4 28;70(2):363–74. 10.1016/j.neuron.2011.03.009 21521620

[96] Serino A, Alsmith A, Costantini M, Mandrigin A, Tajadura-Jimenez A, Lopez C. Bodily ownership and self-location: components of bodily self-consciousness. Consciousness and Cognition. 2013 12;22(4):1239–52. 10.1016/j.concog.2013.08.013 24025475

[97] Tupper KW, Labate BC. Ayahuasca, Psychedelic Studies and Health Sciences: The Politics ofKnowledge and Inquiry into an Amazonian Plant Brew. Current drug abuse reviews. 2014 12 pp. 1–10. 25563448 10.2174/1874473708666150107155042

